# Supplementary material for: Prioritization of Fluorescence In Situ Hybridization (FISH) Probes for Differentiating Primary Sites of Neuroendocrine Tumors with Machine Learning
Source: Int J Mol Sci. 2023 Dec 12;24(24):17401. doi: 10.3390/ijms242417401 (PMC10743810; doi:10.3390/ijms242417401)
Supplement: Supplementary file 1 [file ijms-24-17401-s001.zip › ijms-2722010-supplementary.pdf]

Supplementary Figures and Tables

Supplementary Table S1. Models and parameter values for hyperparameter tuning. Parameters were tuned with a grid search and 10-fold CV with training sets.

**DT**	
**Parameter**	**Values**
mincut	2,3,4,5,6,7,8
minsize	5,7,10,12,15
mindev	0.1,0.01,0.001
split	gini, "deviance"
best	2,4,5,6,7,8,9,10,12,15
k	0,1,2,3,4,5,6,7,8,9,10
**RF**	
**Parameter**	**Values**
mtry	2,8,13,18,24
**lasso**	
**Parameter**	**Values**
alpha	1
lambda	0.000019,0.00019,0.0019,0.019,0.19
**NB**	
**Parameter**	**Values**
laplace	0
**SVM-L**	
**Parameter**	**Values**
C	0.0625, 0.184, 0.54, 1.59, 4.67, 13.7, 40.3, 119, 348, 1024
**SVM-P**	
**Parameter**	**Values**
C	0.05,0.25,1,4,16,20
degree	1,2,3,4,5
scale	0.001,0.0012,0.015,0.17,1,2
**SVM-RB**	
**Parameter**	**Values**
C	0.05,0.25,1,4,16,20
sigma	0.01,0.03,0.05,0.07,0.09,0.1
**XGBTree**	
**Parameter**	**Values**
nrounds	50,100,150,200,250
max_depth	1,2,3,4,5
**Elasticnet**	
**Parameter**	**Values**
alpha	0.1,0.325,0.55,0.775,1
lambda	0.000019,0.00019,0.0019,0.019,0.19

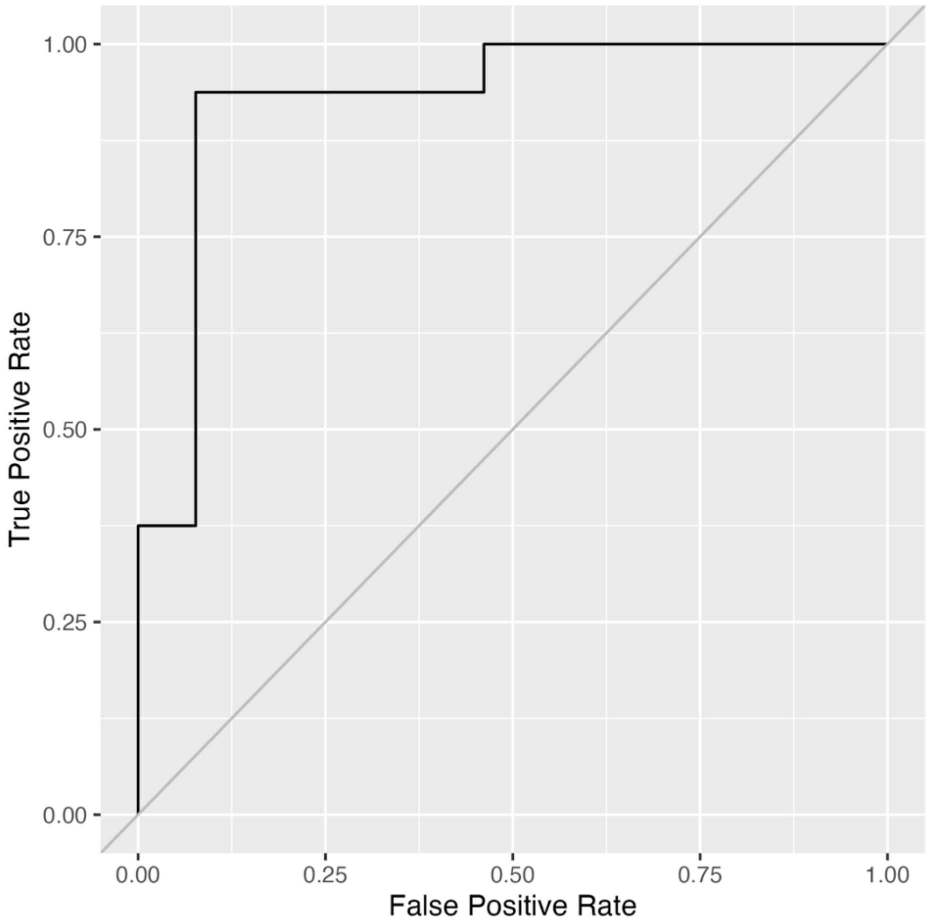

Supplementary Figure S1. ROC curve for Naïve bayes model trained on raw dataset. AUROC curve = 0.928.

Supplementary Table S2. Parameters of top models from top performing datasets.

**Top Models - Raw Dataset**					
	laplace				
NB	0				
	nrounds	max depth			
XGBTree	50	5			
**Top Models - Median-Imputed Dataset**					
	C	sigma			
SVM-RB	4	0.01			
	mincut	minsize	mindev	split	pruning parameter (best or k)
DT	5	10	0.01	gini	best=10
**Top DT Models from Manual Monte Carlo CV - KNN-Imputed Data with Single Categorical Variable Transformation**					
**Decision Tree Models**	**mincut**	**minsize**	**mindev**	**split**	**pruning parameter (best or k)**
Training Set 1	6	15	0.01	deviance	best=10
Training Set 2	5	10	0.01	deviance	best=4
Training Set 3	5	15	0.01	gini	k=2

Training Set 4	6	12	0.1	gini	best=4
Training Set 5	6	15	0.01	deviance	k=3
Training Set 6	5	12	0.001	deviance	best=15
Training Set 7	2	5	0.1	gini	best=2
Training Set 8	2	5	0.1	gini	k=8
Training Set 9	3	15	0.1	gini	k=1
Training Set 10	2	5	0.1	gini	best=2
**Full Dataset DT model**					
	**mincut**	**minsize**	**mindev**	**split**	**pruning parameter**
Full Dataset	4	10	0.01	deviance	best=4

Supplementary Table S3. Test set performance metrics for all models on four imputed datasets. Imputation methods used were impute by mean, median, KNN, and bagged trees models.

**Impute Mean**						
	**Brier Score**	**Accuracy**	**Kappa**	**AUROC Curve**	**Sensitivity**	**Specificity**
DT	0.265	65.5%	0.322	0.692	0.563	0.769
RF	0.096	86.2%	0.725	0.959	0.813	0.923
lasso	0.165	75.9%	0.494	0.822	0.938	0.539
NB	0.105	89.7%	0.789	0.966	0.938	0.846
SVM-L	0.136	86.2%	0.717	0.875	0.938	0.769
SVM-P	0.100	86.2%	0.717	0.904	0.938	0.769
SVM-RB	0.069	93.1%	0.861	0.981	0.938	0.923
XGBTree	0.145	79.3%	0.576	0.880	0.875	0.692
Elasticnet	0.148	82.8%	0.644	0.889	0.938	0.692
Log Reg	0.069	93.1%	0.861	0.981	0.938	0.923
**Impute Median**						
	**Brier Score**	**Accuracy**	**Kappa**	**AUROC Curve**	**Sensitivity**	**Specificity**
DT	0.183	72.4%	0.442	0.820	0.750	0.692
RF	0.095	86.2%	0.721	0.971	0.875	0.846
lasso	0.139	75.9%	0.494	0.914	0.938	0.539
NB	0.102	89.7%	0.792	0.942	0.875	0.923
SVM-L	0.138	82.8%	0.649	0.894	0.875	0.769
SVM-P	0.095	89.7%	0.789	0.909	0.938	0.846
SVM-RB	0.066	93.1%	0.861	0.986	0.938	0.923
XGBTree	0.098	89.7%	0.792	0.923	0.875	0.923
Elasticnet	0.122	82.8%	0.644	0.923	0.938	0.692
Log Reg	0.066	93.1%	0.861	0.986	0.938	0.923
**Impute KNN**						
	**Brier Score**	**Accuracy**	**Kappa**	**AUROC Curve**	**Sensitivity**	**Specificity**
DT	0.200	72.4%	0.450	0.776	0.688	0.769
RF	0.110	86.2%	0.721	0.918	0.875	0.846
lasso	0.144	75.9%	0.501	0.885	0.875	0.615

NB	0.105	89.7%	0.792	0.952	0.875	0.923
SVM-L	0.132	82.8%	0.649	0.904	0.875	0.769
SVM-P	0.108	86.2%	0.721	0.904	0.875	0.846
SVM-RB	0.077	89.7%	0.792	0.976	0.875	0.923
XGBTree	0.167	75.9%	0.516	0.822	0.750	0.769
Elasticnet	0.140	75.9%	0.501	0.885	0.875	0.615
Log Reg	0.077	89.7%	0.792	0.976	0.875	0.923
**Impute Bagged Tree Models**						
	**Brier Score**	**Accuracy**	**Kappa**	**AUROC Curve**	**Sensitivity**	**Specificity**
DT	0.206	69.0%	0.359	0.745	0.813	0.539
RF	0.102	89.7%	0.789	0.947	0.938	0.846
lasso	0.165	79.3%	0.569	0.817	0.938	0.615
NB	0.103	89.7%	0.792	0.986	0.875	0.923
SVM-L	0.121	86.2%	0.717	0.880	0.938	0.769
SVM-P	0.104	89.7%	0.789	0.923	0.938	0.846
SVM-RB	0.068	93.1%	0.861	0.962	0.938	0.923
XGBTree	0.131	75.9%	0.516	0.904	0.750	0.769
Elasticnet	0.152	79.3%	0.569	0.841	0.938	0.615
Log Reg	0.068	93.1%	0.861	0.962	0.938	0.923

Supplementary Table S4. Mean 10-fold CV training set performance metrics for all models on four imputed datasets with a transformation of the data to single categorical variables for each biomarker. Imputation methods used were impute by mean, median, KNN, and bagged trees models.

**Impute Mean**						
	**Brier Score**	**Accuracy**	**Kappa**	**AUROC Curve**	**Sensitivity**	**Specificity**
DT	0.184	71.2%	0.395	0.776	0.755	0.645
RF	0.174	77.2%	0.499	0.797	0.898	0.580
lasso	0.186	77.2%	0.471	0.710	0.955	0.490
NB	0.193	75.6%	0.453	0.721	0.912	0.515
SVM-L	0.180	75.6%	0.424	0.787	0.969	0.430
SVM-P	0.179	76.4%	0.443	0.784	0.969	0.450
SVM-RB	0.178	75.6%	0.426	0.763	0.955	0.450
XGBTree	0.177	72.1%	0.414	0.784	0.767	0.650
Elasticnet	0.186	77.2%	0.471	0.710	0.955	0.490
Log Reg	0.199	70.6%	0.362	0.754	0.798	0.560
**Impute Median**						
	**Brier Score**	**Accuracy**	**Kappa**	**AUROC Curve**	**Sensitivity**	**Specificity**
DT	0.186	73.7%	0.420	0.771	0.864	0.540
RF	0.187	76.6%	0.474	0.766	0.898	0.560
lasso	0.180	77.4%	0.484	0.770	0.941	0.520
NB	0.192	75.7%	0.466	0.752	0.883	0.565
SVM-L	0.171	78.2%	0.499	0.754	0.955	0.520

SVM-P	0.170	77.4%	0.484	0.811	0.941	0.520
SVM-RB	0.168	77.4%	0.484	0.843	0.941	0.520
XGBTree	0.177	76.6%	0.469	0.778	0.926	0.520
Elasticnet	0.180	77.4%	0.484	0.770	0.941	0.520
Log Reg	0.202	72.4%	0.395	0.750	0.841	0.540
**Impute KNN**						
	**Brier Score**	**Accuracy**	**Kappa**	**AUROC Curve**	**Sensitivity**	**Specificity**
DT	0.180	78.2%	0.524	0.781	0.883	0.630
RF	0.176	74.0%	0.443	0.806	0.812	0.630
lasso	0.172	79.1%	0.543	0.797	0.883	0.650
NB	0.205	74.1%	0.440	0.806	0.843	0.590
SVM-L	0.169	79.1%	0.532	0.801	0.926	0.590
SVM-P	0.168	79.1%	0.532	0.790	0.926	0.590
SVM-RB	0.168	79.1%	0.537	0.786	0.912	0.610
XGBTree	0.175	79.1%	0.543	0.799	0.883	0.650
Elasticnet	0.172	79.1%	0.543	0.797	0.883	0.650
Log Reg	0.210	71.3%	0.384	0.754	0.812	0.565
**Impute Bagged Tree Models**						
	**Brier Score**	**Accuracy**	**Kappa**	**AUROC Curve**	**Sensitivity**	**Specificity**
DT	0.209	71.3%	0.375	0.723	0.826	0.545
RF	0.211	73.1%	0.395	0.726	0.883	0.495
lasso	0.192	77.5%	0.492	0.728	0.926	0.545
NB	0.204	74.1%	0.442	0.756	0.841	0.590
SVM-L	0.179	77.5%	0.492	0.744	0.926	0.545
SVM-P	0.179	77.5%	0.492	0.759	0.926	0.545
SVM-RB	0.181	77.5%	0.492	0.716	0.926	0.545
XGBTree	0.191	77.5%	0.492	0.740	0.926	0.545
Elasticnet	0.192	77.5%	0.492	0.728	0.926	0.545
Log Reg	0.206	70.7%	0.355	0.757	0.841	0.500

Supplementary Table S5. Test set performance metrics for all models on four imputed datasets with a transformation of the data to single categorical variables for each biomarker. Imputation methods used were impute by mean, median, KNN, and bagged trees models.

**Impute Mean**						
	**Brier Score**	**Accuracy**	**Kappa**	**AUROC Curve**	**Sensitivity**	**Specificity**
DT	0.212	65.5%	0.293	0.760	0.750	0.539
RF	0.147	75.9%	0.494	0.885	0.938	0.539
lasso	0.199	72.4%	0.417	0.755	0.938	0.462
NB	0.175	75.9%	0.494	0.793	0.938	0.539
SVM-L	0.189	72.4%	0.417	0.798	0.938	0.462
SVM-P	0.186	72.4%	0.417	0.798	0.938	0.462
SVM-RB	0.173	72.4%	0.417	0.880	0.938	0.462

XGBTree	0.180	75.9%	0.494	0.813	0.938	0.539
Elasticnet	0.199	72.4%	0.417	0.755	0.938	0.462
Log Reg	0.173	72.4%	0.417	0.880	0.938	0.462
**Impute Median**						
	**Brier Score**	**Accuracy**	**Kappa**	**AUROC Curve**	**Sensitivity**	**Specificity**
DT	0.218	72.4%	0.417	0.668	0.938	0.462
RF	0.133	79.3%	0.569	0.930	0.938	0.615
lasso	0.177	75.9%	0.494	0.858	0.938	0.539
NB	0.172	75.9%	0.501	0.839	0.875	0.615
SVM-L	0.182	72.4%	0.417	0.772	0.938	0.462
SVM-P	0.163	75.9%	0.494	0.858	0.938	0.539
SVM-RB	0.158	79.3%	0.569	0.916	0.938	0.615
XGBTree	0.170	75.9%	0.494	0.815	0.938	0.539
Elasticnet	0.177	75.9%	0.494	0.858	0.938	0.539
Log Reg	0.158	79.3%	0.569	0.916	0.938	0.615
**Impute KNN**						
	**Brier Score**	**Accuracy**	**Kappa**	**AUROC Curve**	**Sensitivity**	**Specificity**
DT	0.157	82.8%	0.654	0.841	0.813	0.846
RF	0.154	79.3%	0.582	0.873	0.813	0.769
lasso	0.177	75.9%	0.509	0.803	0.813	0.692
NB	0.180	79.3%	0.582	0.861	0.813	0.769
SVM-L	0.174	75.9%	0.509	0.808	0.813	0.692
SVM-P	0.176	75.9%	0.509	0.793	0.813	0.692
SVM-RB	0.169	79.3%	0.582	0.817	0.813	0.769
XGBTree	0.183	75.9%	0.509	0.781	0.813	0.692
Elasticnet	0.177	75.9%	0.509	0.803	0.813	0.692
Log Reg	0.169	79.3%	0.582	0.817	0.813	0.769
**Impute Bagged Tree Models**						
	**Brier Score**	**Accuracy**	**Kappa**	**AUROC Curve**	**Sensitivity**	**Specificity**
DT	0.205	75.9%	0.494	0.671	0.938	0.539
RF	0.132	79.3%	0.563	0.916	1.000	0.539
lasso	0.204	72.4%	0.417	0.704	0.938	0.462
NB	0.175	72.4%	0.426	0.858	0.875	0.539
SVM-L	0.200	72.4%	0.417	0.762	0.938	0.462
SVM-P	0.200	72.4%	0.417	0.829	0.938	0.462
SVM-RB	0.198	72.4%	0.417	0.839	0.938	0.462
XGBTree	0.196	72.4%	0.417	0.743	0.938	0.462
Elasticnet	0.204	72.4%	0.417	0.704	0.938	0.462
Log Reg	0.198	72.4%	0.417	0.839	0.938	0.462

Supplementary Table S6. Counts and probabilities of FISH test results across samples with KNN-imputed data.

**Counts of FISH Test Results**								
	ERBB2	***CKS1B***	***FGFR3***	***CSF1R***	***MET***	***CDKN2A***	***SMAD4***	***CCNE1***
Loss	26	87	15	11	38	71	117	9
Normal	72	47	25	67	47	37	2	40
Gain	46	10	104	66	59	36	25	95
**Probability of FISH Test Result**								
	ERBB2	***CKS1B***	***FGFR3***	***CSF1R***	***MET***	***CDKN2A***	***SMAD4***	***CCNE1***
Loss	0.181	0.604	0.104	0.076	0.264	0.493	0.813	0.063
Normal	0.500	0.326	0.174	0.465	0.326	0.257	0.014	0.278
Gain	0.319	0.069	0.722	0.458	0.410	0.250	0.174	0.660

Supplementary Table S7. Percentages of sbNET samples in the full dataset and ten training sets from the ten random 80/20 splits for Monte Carlo CV.

	**All Data**	**Fold 1**	**Fold 2**	**Fold 3**	**Fold 4**	**Fold 5**	**Fold 6**	**Fold 7**	**Fold 8**	**Fold 9**	**Fold 10**
sbNET training set percentage	59.0%	60.0%	58.3%	59.1%	58.3%	64.4%	57.4%	53.9%	58.3%	62.6%	58.3%

(a)

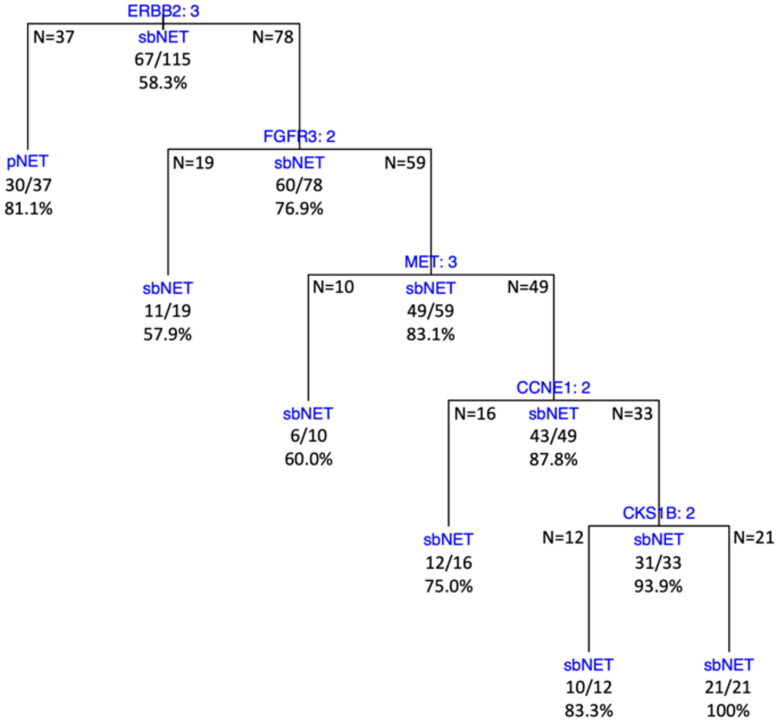

(**b**)

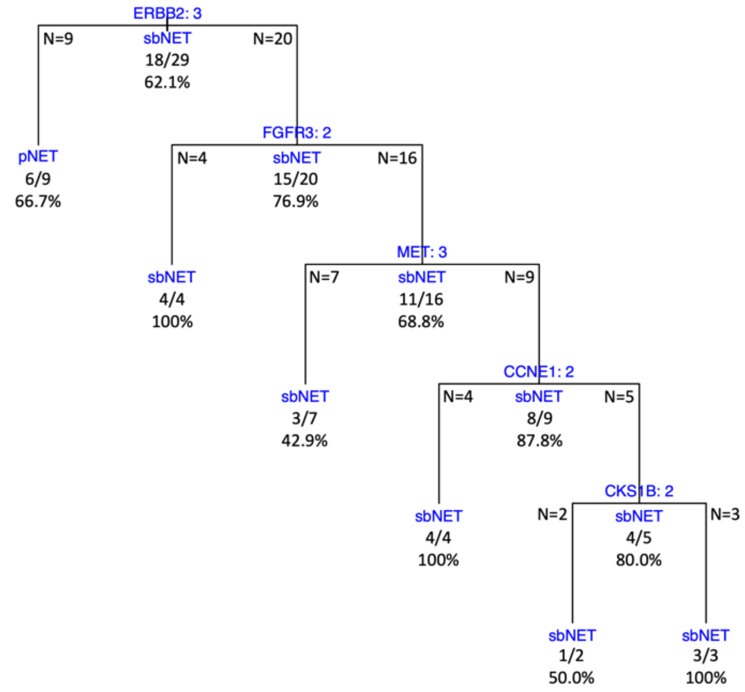
ERBB2: 3
N=9
sbNET
18/29
62.1%
N=20
pNET
6/9
66.7%
FGFR3: 2
N=4
sbNET
15/20
76.9%
N=16
sbNET
4/4
100%
MET: 3
N=7
sbNET
11/16
68.8%
N=9
sbNET
3/7
42.9%
CCNE1: 2
N=4
sbNET
8/9
87.8%
N=5
sbNET
4/4
100%
CKS1B: 2
N=2
sbNET
4/5
80.0%
N=3
sbNET
1/2
50.0%
sbNET
3/3
100%

(**c**)

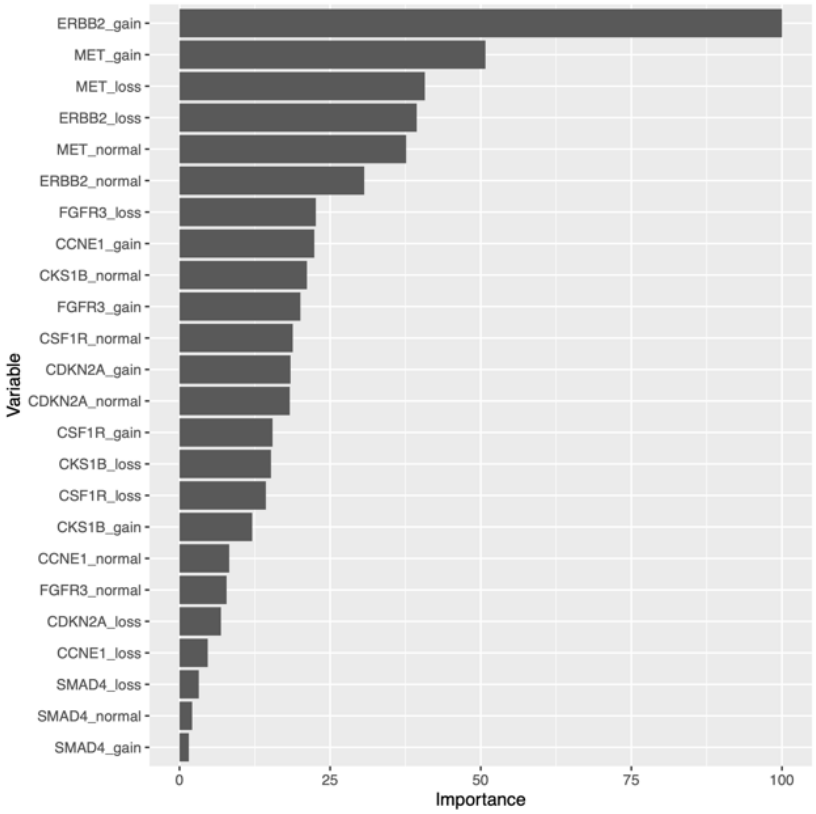
ERBB2_gain
MET_gain
MET_loss
ERBB2_loss
MET_normal
ERBB2_normal
FGFR3_loss
CCNE1_gain
CKS1B_normal
FGFR3_gain
CSF1R_normal
CDKN2A_gain
CDKN2A_normal
CSF1R_gain
CKS1B_loss
CSF1R_loss
CKS1B_gain
CCNE1_normal
FGFR3_normal
CDKN2A_loss
CCNE1_loss
SMAD4_loss
SMAD4_normal
SMAD4_gain
Variable
0
25
50
75
100
Importance

(d)

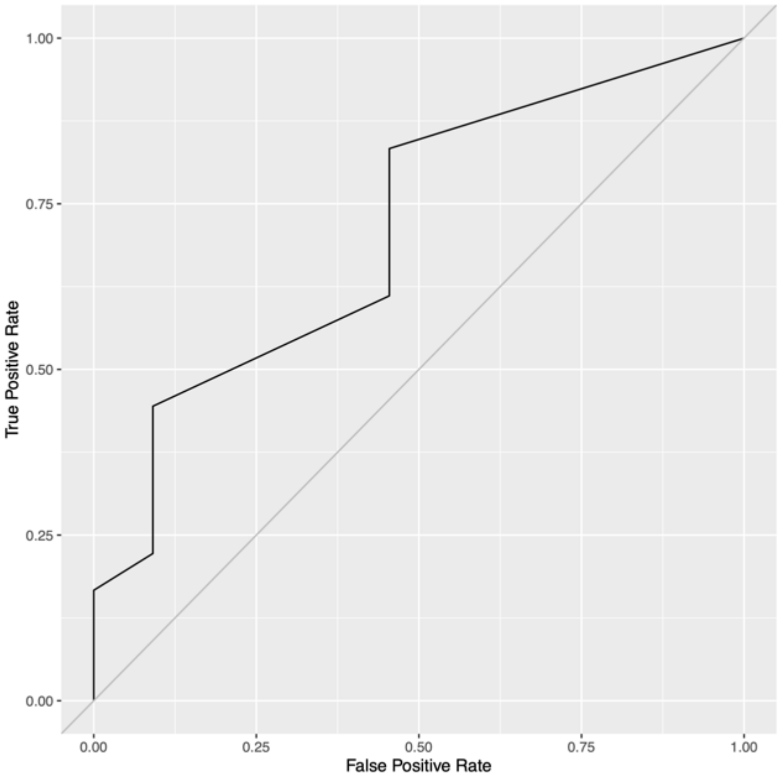

Supplementary Figure S2. DT model trained on KNN-imputed data with a transformation of the data to single categorical variables for each biomarker. Training and test sets constructed with random 80/20 dataset split (Training and Test Set 2) for manual Monte Carlo CV. (**a**) Training set DT model plot with internal nodes shown with variables being split with splitting criteria of 1 = loss, 2 = normal, and 3 = gain for number of copies at the genomic regions. Samples with a variable value equal to the splitting criteria are split down the left branch and values different than the splitting criteria are split down the right branch. Terminal nodes show the final classification of the samples with either pNET or sbNET. Classification of sample subsets at internal nodes are shown under horizontal lines and sample numbers of subsets from the split are displayed at the edges of the horizontal lines. Each node lists correctly classified samples as a fraction and percent accuracy. Tree accuracy = 78.3% (90/115) (**b**) Test set DT model plot with same design as (a) with test set data. Tree accuracy = 72.4% (21/29). (**c**) Permutation-based variable importance. All variables included in the model are assessed. Permutations per variable were performed 25 times and performance is assessed based on mean brier score. (**d**) ROC curve. AUROC curve = 0.710.

(**a**)

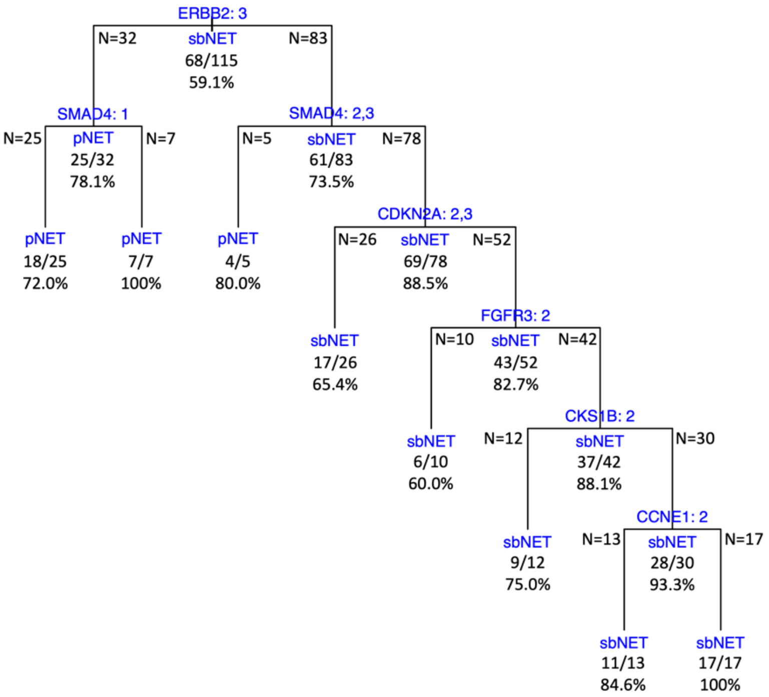
ERBB2: 3
N=32
sbNET
N=83
68/115
59.1%
SMAD4: 1
N=25
pNET
N=7
25/32
78.1%
SMAD4: 2,3
N=5
sbNET
N=78
61/83
73.5%
pNET
18/25
72.0%
pNET
7/7
100%
pNET
4/5
80.0%
CDKN2A: 2,3
N=26
sbNET
N=52
69/78
88.5%
sbNET
17/26
65.4%
FGFR3: 2
N=10
sbNET
N=42
43/52
82.7%
sbNET
6/10
60.0%
CKS1B: 2
N=12
sbNET
N=30
37/42
88.1%
sbNET
9/12
75.0%
CCNE1: 2
N=13
sbNET
N=17
28/30
93.3%
sbNET
11/13
84.6%
sbNET
17/17
100%

(**b**)

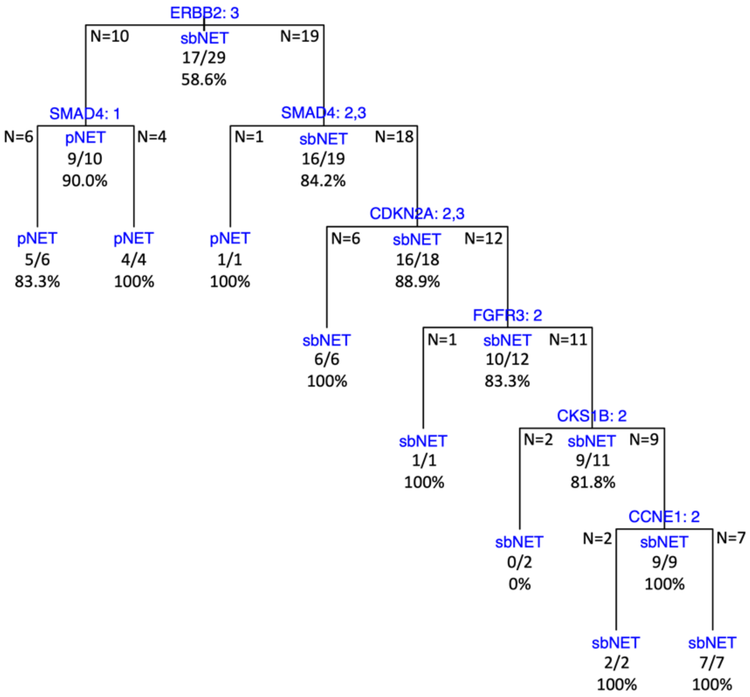
ERBB2: 3
N=10
sbNET
N=19
17/29
58.6%
SMAD4: 1
N=6
pNET
N=4
9/10
90.0%
SMAD4: 2,3
N=1
sbNET
N=18
16/19
84.2%
pNET
5/6
83.3%
pNET
4/4
100%
pNET
1/1
100%
CDKN2A: 2,3
N=6
sbNET
N=12
16/18
88.9%
sbNET
6/6
100%
FGFR3: 2
N=1
sbNET
N=11
10/12
83.3%
sbNET
1/1
100%
CKS1B: 2
N=2
sbNET
N=9
9/11
81.8%
sbNET
0/2
0%
CCNE1: 2
N=2
sbNET
N=7
9/9
100%
sbNET
2/2
100%
sbNET
7/7
100%

(c)

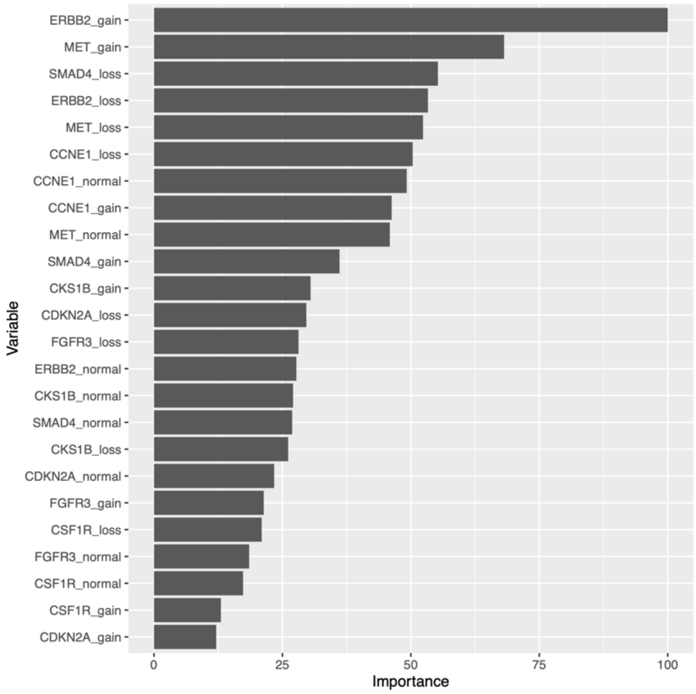

(d)

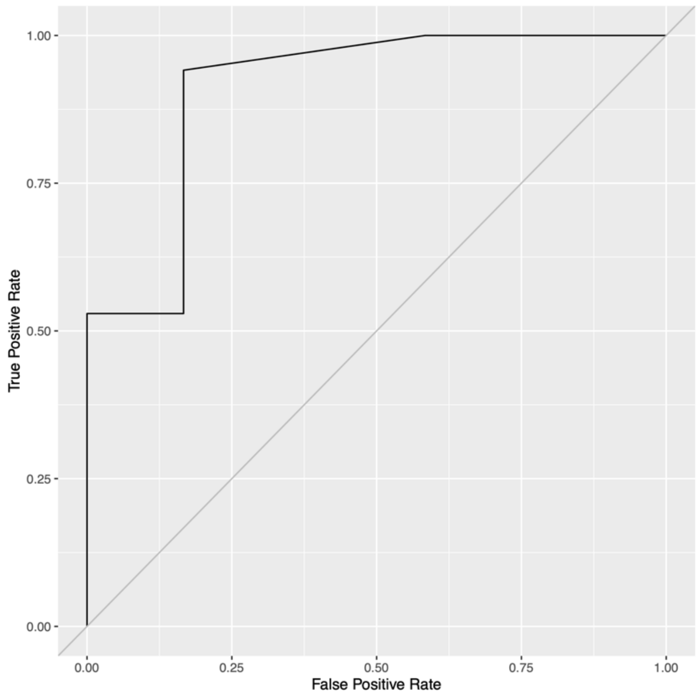

Supplementary Figure S3. DT model trained on KNN-imputed data with a transformation of the data to single categorical variables for each biomarker. Training and test sets constructed with random 80/20 dataset split (Training and Test Set 3) for manual Monte Carlo CV. (**a**) Training set DT model plot with internal nodes shown with variables being split with splitting criteria of 1 = loss, 2 = normal, and 3 = gain for number of copies at the genomic regions. Samples with a variable value equal to the splitting criteria are split down the left branch and values different than the splitting criteria are split down the right branch. Terminal nodes show the final classification of the samples with either pNET or sbNET. Classification of sample subsets at internal nodes are shown under horizontal lines and sample numbers of subsets from the split are displayed at the edges of the horizontal lines. Each node lists correctly classified samples as a fraction and percent accuracy. Tree accuracy = 77.4% (89/115) (**b**) Test set DT model plot with same design as (a) with test set data. Tree accuracy = 89.7% (26/29) (**c**) Permutation-based variable importance. All variables included in the model are assessed. Permutations per variable were performed 25 times and performance is assessed based on mean brier score. (**d**) ROC curve. AUROC curve = 0.909.

(**a**)

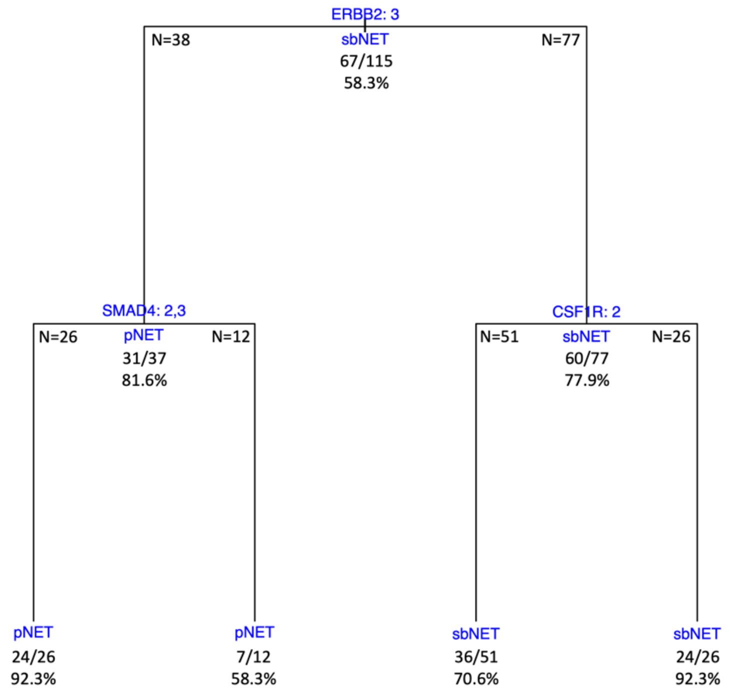
ERBB2: 3
sbNET
67/115
58.3%
N=38
N=77
SMAD4: 2,3
pNET
31/37
81.6%
N=26
N=12
CSF1R: 2
sbNET
60/77
77.9%
N=51
N=26
pNET
24/26
92.3%
pNET
7/12
58.3%
sbNET
36/51
70.6%
sbNET
24/26
92.3%

(**b**)

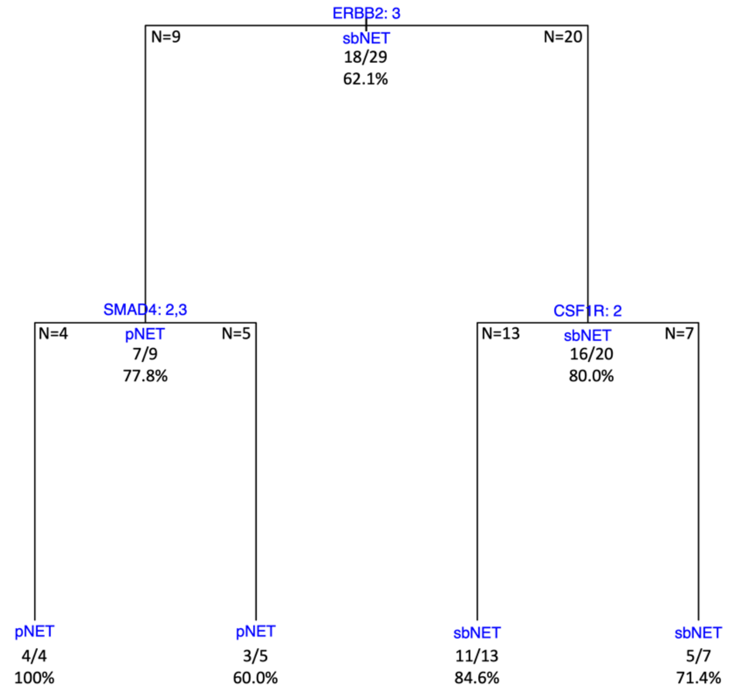
ERBB2: 3
sbNET
18/29
62.1%
N=9
N=20
SMAD4: 2,3
pNET
7/9
77.8%
N=4
N=5
CSF1R: 2
sbNET
16/20
80.0%
N=13
N=7
pNET
4/4
100%
pNET
3/5
60.0%
sbNET
11/13
84.6%
sbNET
5/7
71.4%

(c)

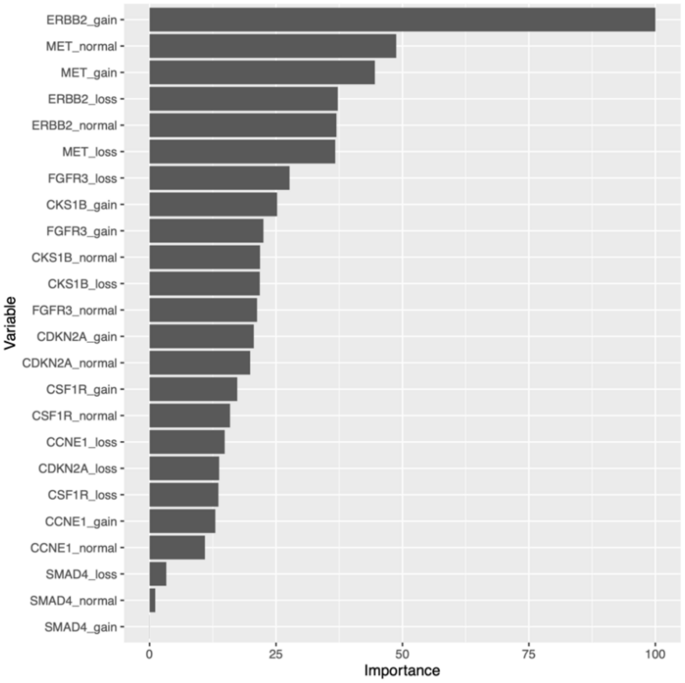

(d)

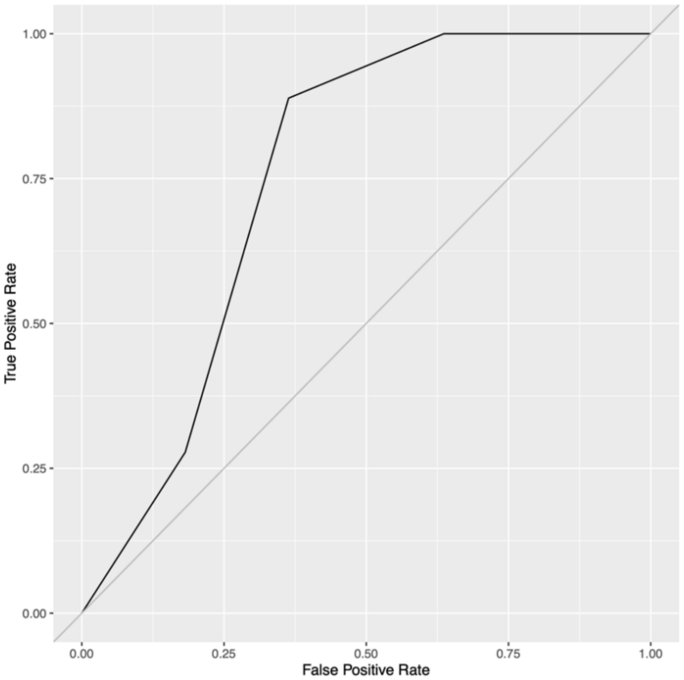

Supplementary Figure S4. DT model trained on KNN-imputed data with a transformation of the data to single categorical variables for each biomarker. Training and test sets constructed with random 80/20 dataset split (Training and Test Set 4) for manual Monte Carlo CV. (**a**) Training set DT model plot with internal nodes shown with variables being split with splitting criteria of 1 = loss, 2 = normal, and 3 = gain for number of copies at the genomic regions. Samples with a variable value equal to the splitting criteria are split down the left branch and values different than the splitting criteria are split down the right branch. Terminal nodes show the final classification of the samples with either pNET or sbNET. Classification of sample subsets at internal nodes are shown under horizontal lines and sample numbers of subsets from the split are displayed at the edges of the horizontal lines. Each node lists correctly classified samples as a fraction and percent accuracy. Tree accuracy = 79.1% (91/115). (**b**) Test set DT model plot with same design as (a) with test set data. Tree accuracy = 79.3% (23/29). (**c**) Permutation-based variable importance. All variables included in the model are assessed. Permutations per variable were performed 25 times and performance is assessed based on mean brier score. (**d**) ROC curve. AUROC curve = 0.753.

(a)

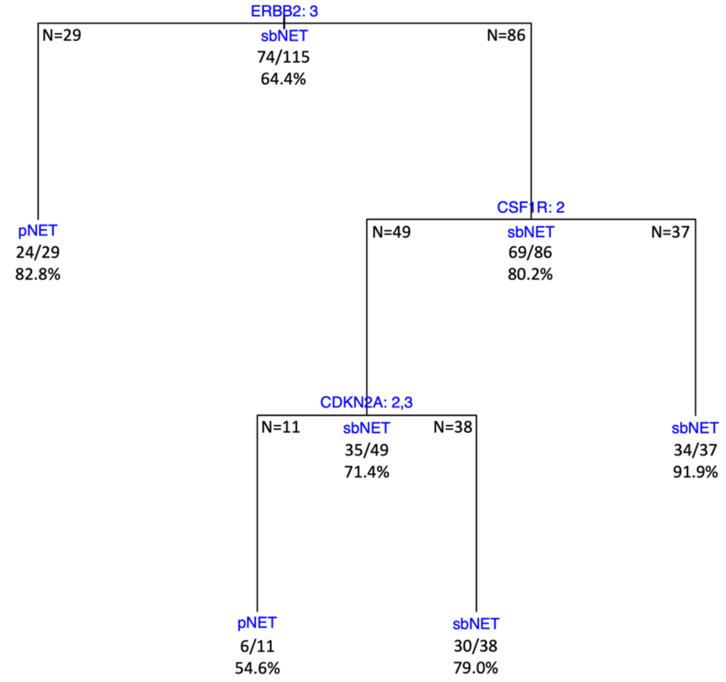
ERBB2: 3
sbNET
74/115
64.4%
N=29
N=86
pNET
24/29
82.8%
CSF1R: 2
sbNET
69/86
80.2%
N=49
N=37
CDKN2A: 2,3
sbNET
35/49
71.4%
N=11
N=38
sbNET
34/37
91.9%
pNET
6/11
54.6%
sbNET
30/38
79.0%

(b)

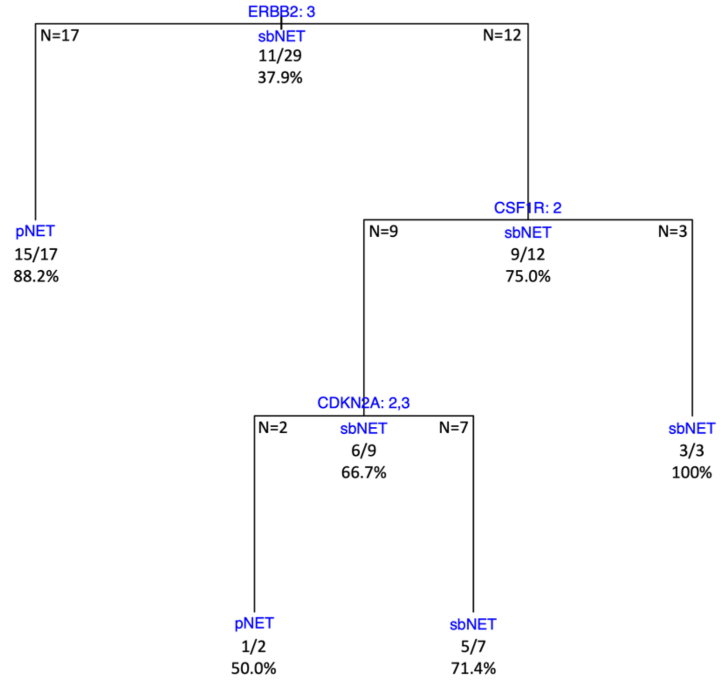
ERBB2: 3
sbNET
11/29
37.9%
N=17
N=12
pNET
15/17
88.2%
CSF1R: 2
sbNET
9/12
75.0%
N=9
N=3
CDKN2A: 2,3
sbNET
6/9
66.7%
N=2
N=7
sbNET
3/3
100%
pNET
1/2
50.0%
sbNET
5/7
71.4%

(c)

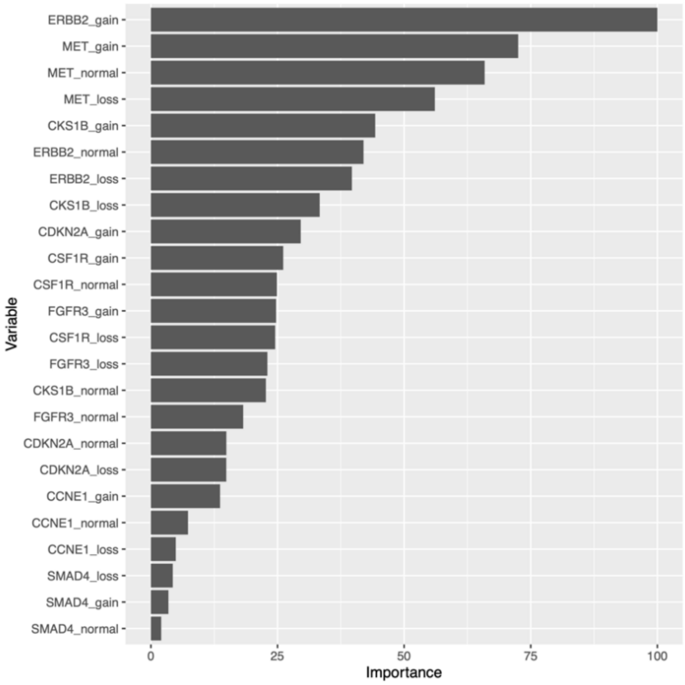

(d)

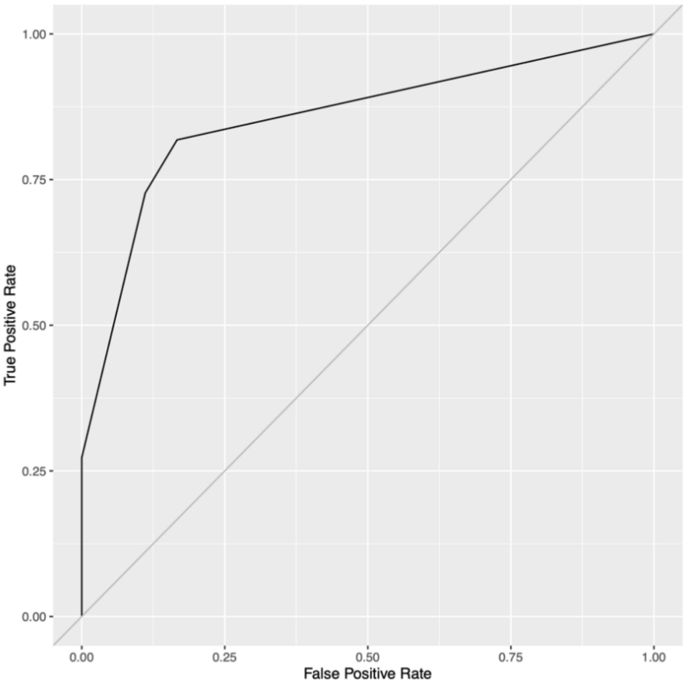

Supplementary Figure S5. DT model trained on KNN-imputed data with a transformation of the data to single categorical variables for each biomarker. Training and test sets constructed with random 80/20 dataset split (Training and Test Set 5) for manual Monte Carlo CV. (**a**) Training set DT model plot with internal nodes shown with variables being split with splitting criteria of 1 = loss, 2 = normal, and 3 = gain for number of copies at the genomic regions. Samples with a variable value equal to the splitting criteria are split down the left branch and values different than the splitting criteria are split down the right branch. Terminal nodes show the final classification of the samples with either pNET or sbNET. Classification of sample subsets at internal nodes are shown under horizontal lines and sample numbers of subsets from the split are displayed at the edges of the horizontal lines. Each node lists correctly classified samples as a fraction and percent accuracy. Tree accuracy = 81.7% (94/115) (**b**) Test set DT model plot with same design as (a) with test set data. Tree accuracy = 82.8% (24/29) (**c**) Permutation-based variable importance. All variables included in the model are assessed. Permutations per variable were performed 25 times and performance is assessed based on mean brier score. (**d**) ROC curve. AUROC curve = 0.856.

(**a**)

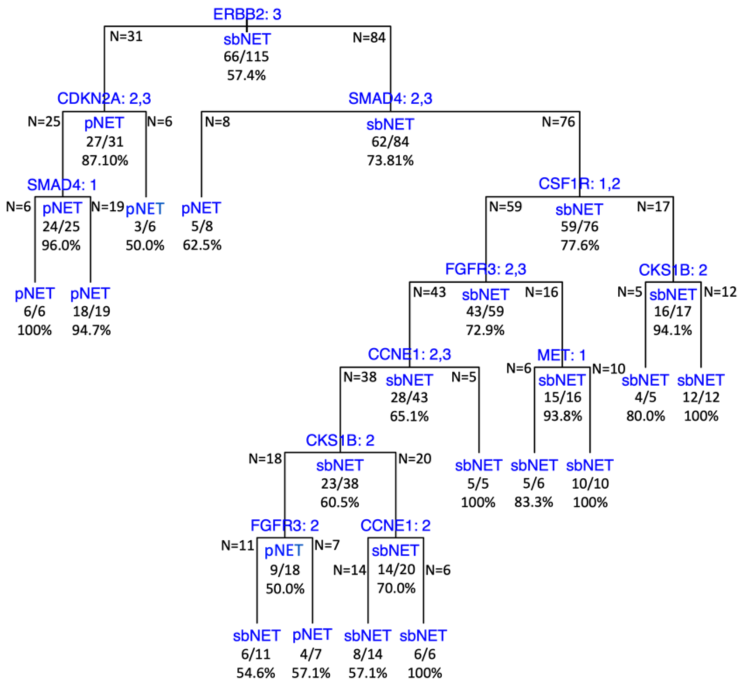
ERBB2: 3
N=31
sbNET
66/115
57.4%
N=84
CDKN2A: 2,3
N=25
pNET
27/31
87.10%
N=6
SMAD4: 2,3
N=8
sbNET
62/84
73.81%
N=76
SMAD4: 1
N=6
pNET
24/25
96.0%
N=19
pNET
3/6
50.0%
pNET
5/8
62.5%
CSF1R: 1,2
N=59
sbNET
59/76
77.6%
N=17
pNET
6/6
100%
pNET
18/19
94.7%
FGFR3: 2,3
N=43
sbNET
43/59
72.9%
N=16
CKS1B: 2
N=5
sbNET
16/17
94.1%
N=12
CCNE1: 2,3
N=38
sbNET
28/43
65.1%
N=5
MET: 1
N=6
sbNET
15/16
93.8%
N=10
sbNET
4/5
80.0%
sbNET
12/12
100%
CKS1B: 2
N=18
sbNET
23/38
60.5%
N=20
sbNET
5/5
100%
sbNET
5/6
83.3%
sbNET
10/10
100%
FGFR3: 2
N=11
pNET
9/18
50.0%
N=7
CCNE1: 2
sbNET
14/20
70.0%
N=14
N=6
sbNET
6/11
54.6%
pNET
4/7
57.1%
sbNET
8/14
57.1%
sbNET
6/6
100%

(**b**)

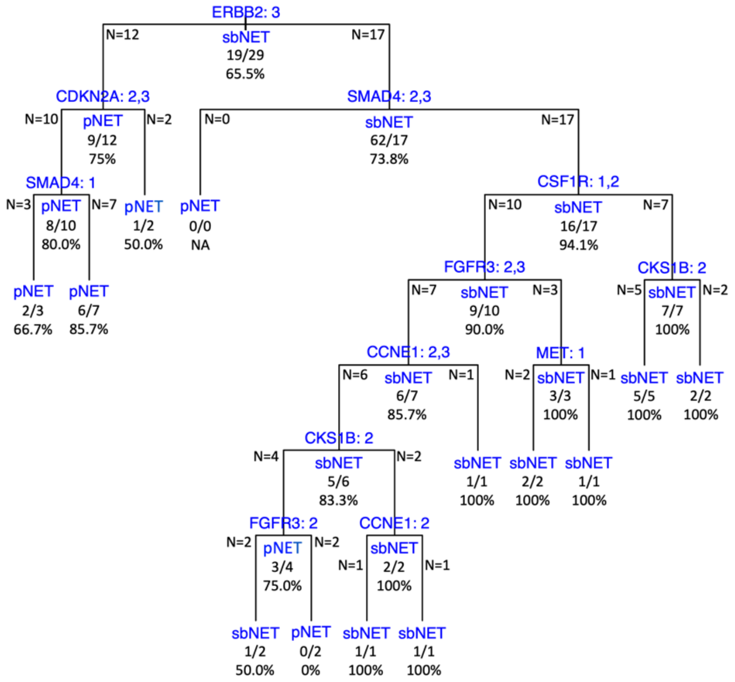
ERBB2: 3
N=12
sbNET
19/29
65.5%
N=17
CDKN2A: 2,3
N=10
pNET
9/12
75%
N=2
SMAD4: 2,3
N=0
sbNET
62/17
73.8%
N=17
SMAD4: 1
N=3
pNET
8/10
80.0%
N=7
pNET
1/2
50.0%
pNET
0/0
NA
CSF1R: 1,2
N=10
sbNET
16/17
94.1%
N=7
pNET
2/3
66.7%
pNET
6/7
85.7%
FGFR3: 2,3
N=7
sbNET
9/10
90.0%
N=3
CKS1B: 2
N=5
sbNET
7/7
100%
N=2
CCNE1: 2,3
N=6
sbNET
6/7
85.7%
N=1
MET: 1
N=2
sbNET
3/3
100%
N=1
sbNET
5/5
100%
sbNET
2/2
100%
CKS1B: 2
N=4
sbNET
5/6
83.3%
N=2
sbNET
1/1
100%
sbNET
2/2
100%
sbNET
1/1
100%
FGFR3: 2
N=2
pNET
3/4
75.0%
N=2
CCNE1: 2
sbNET
2/2
100%
N=1
N=1
sbNET
1/2
50.0%
pNET
0/2
0%
sbNET
1/1
100%
sbNET
1/1
100%

(c)

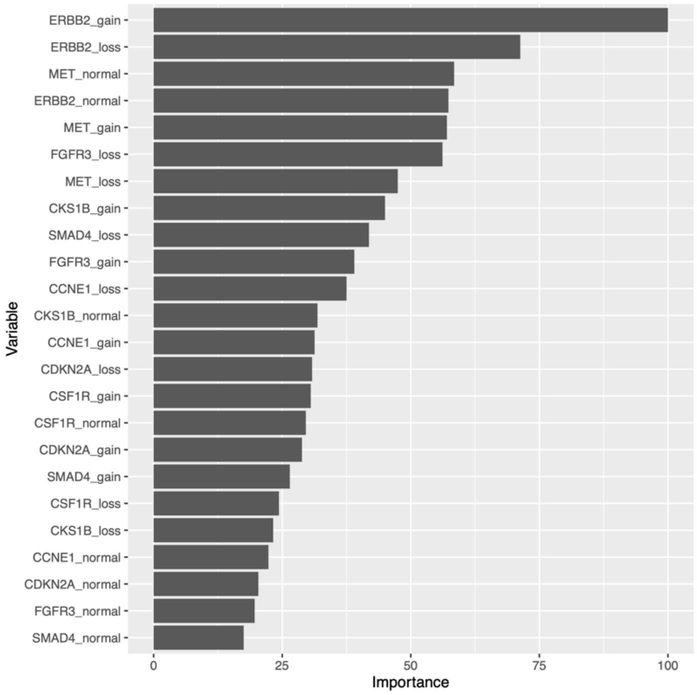

(d)

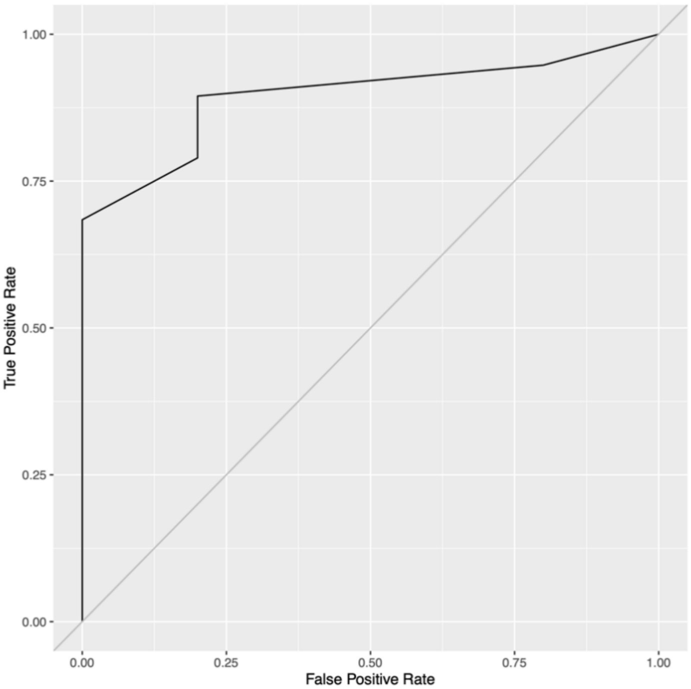

Supplementary Figure S6. DT model trained on KNN-imputed data with a transformation of the data to single categorical variables for each biomarker. Training and test sets constructed with random 80/20 dataset split (Training and Test Set 6) for manual Monte Carlo CV. (**a**) Training set DT model plot with internal nodes shown with variables being split with splitting criteria of 1 = loss, 2 = normal, and 3 = gain for number of copies at the genomic regions. Samples with a variable value equal to the splitting criteria are split down the left branch and values different than the splitting criteria are split down the right branch. Terminal nodes show the final classification of the samples with either pNET or sbNET. Classification of sample subsets at internal nodes are shown under horizontal lines and sample numbers of subsets from the split are displayed at the edges of the horizontal lines. Each node lists correctly classified samples as a fraction and percent accuracy. Tree accuracy = 80.0% (92/115) (**b**) Test set DT model plot with same design as (a) with test set data. Tree accuracy = 79.3% (23/29) (**c**) Permutation-based variable importance. All variables included in the model are assessed. Permutations per variable were performed 25 times and performance is assessed based on mean brier score. (**d**) ROC curve. AUROC curve = 0.895.

(**a**)

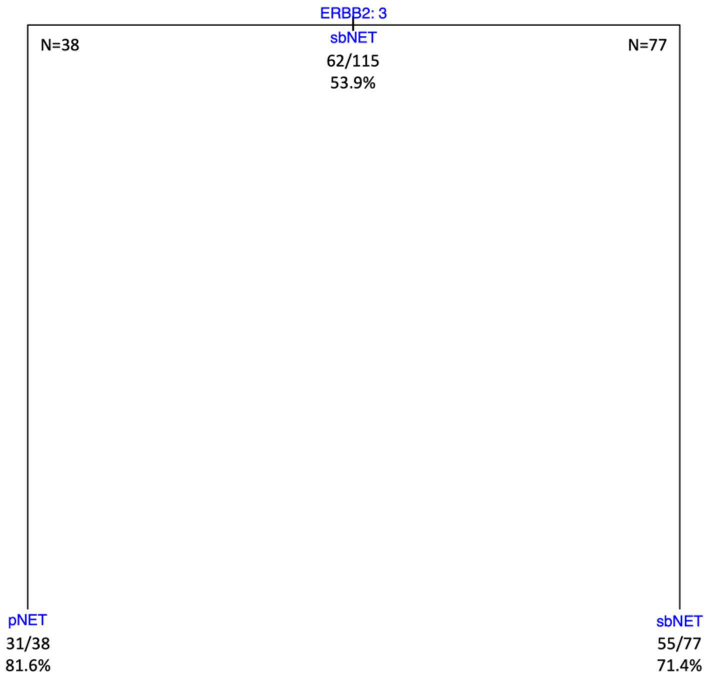
ERBB2: 3
sbNET
62/115
53.9%
N=38
N=77
pNET
31/38
81.6%
sbNET
55/77
71.4%

(**b**)

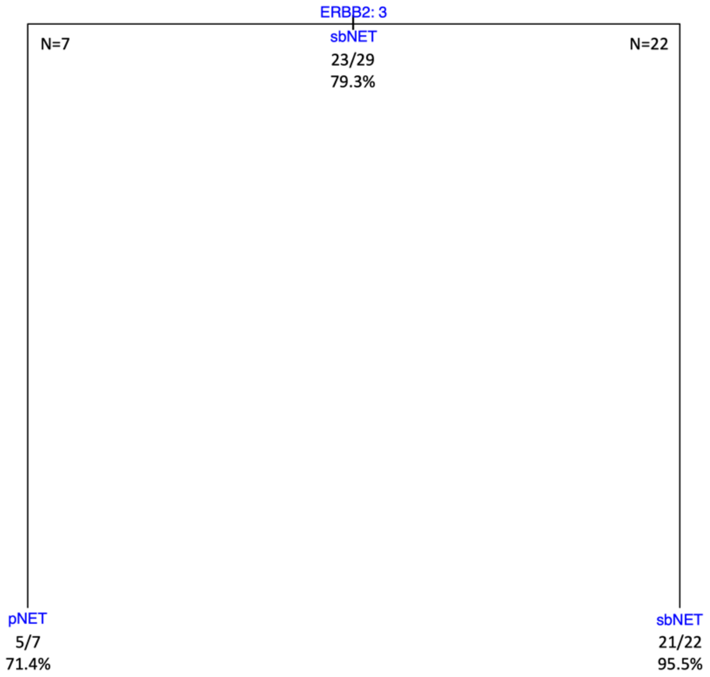
ERBB2: 3
sbNET
23/29
79.3%
N=7
N=22
pNET
5/7
71.4%
sbNET
21/22
95.5%

(c)

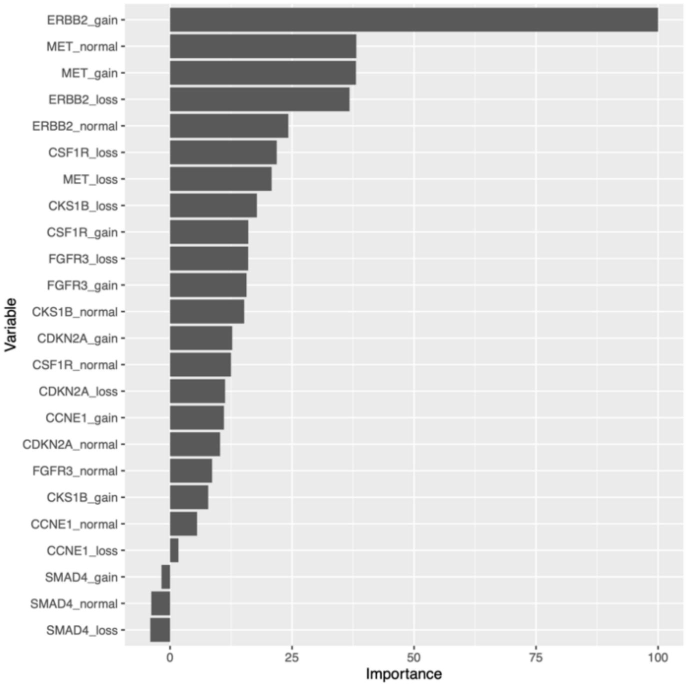

(d)

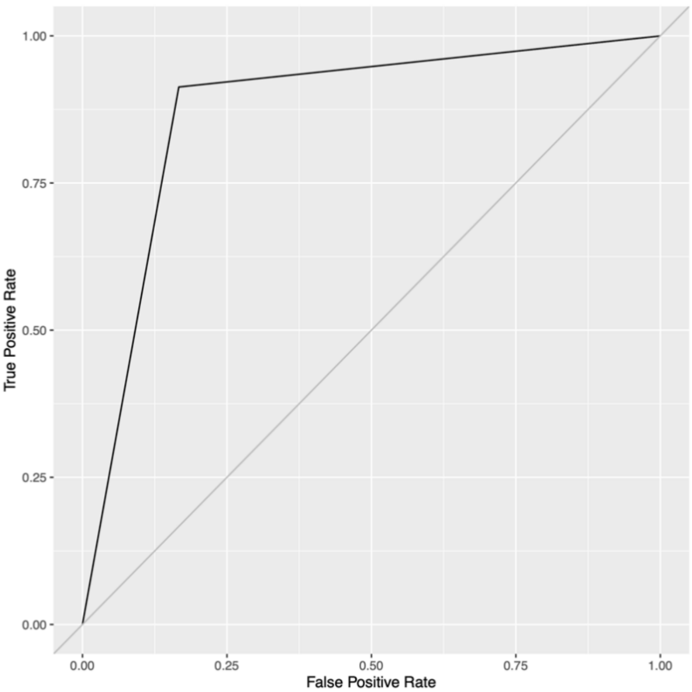

Supplementary Figure S7. DT model trained on KNN-imputed data with a transformation of the data to single categorical variables for each biomarker. Training and test sets constructed with random 80/20 dataset split (Training and Test Set 7) for manual Monte Carlo CV. (**a**) Training set DT model plot with internal nodes shown with variables being split with splitting criteria of 1 = loss, 2 = normal, and 3 = gain for number of copies at the genomic regions. Samples with a variable value equal to the splitting criteria are split down the left branch and values different than the splitting criteria are split down the right branch. Terminal nodes show the final classification of the samples with either pNET or sbNET. Classification of sample subsets at internal nodes are shown under horizontal lines and sample numbers of subsets from the split are displayed at the edges of the horizontal lines. Each node lists correctly classified samples as a fraction and percent accuracy. Tree accuracy = 74.8% (86/115) (**b**) Test set DT model plot with same design as (a) with test set data. Tree accuracy = 89.7% (26/29) (**c**) Permutation-based variable importance. All variables included in the model are assessed. Permutations per variable were performed 25 times and performance is assessed based on mean brier score. (**d**) ROC curve. AUROC curve = 0.873.

(**a**)

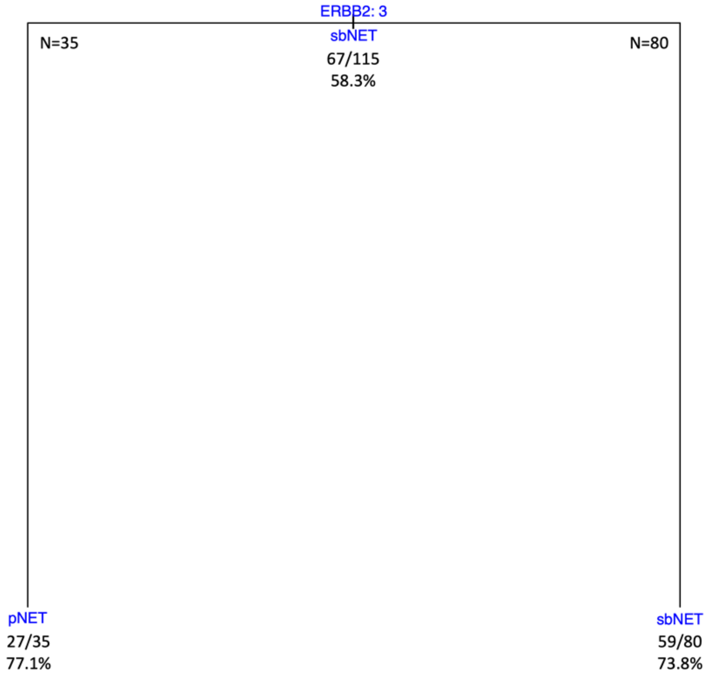
ERBB2: 3
sbNET
67/115
58.3%
N=35
N=80
pNET
27/35
77.1%
sbNET
59/80
73.8%

(**b**)

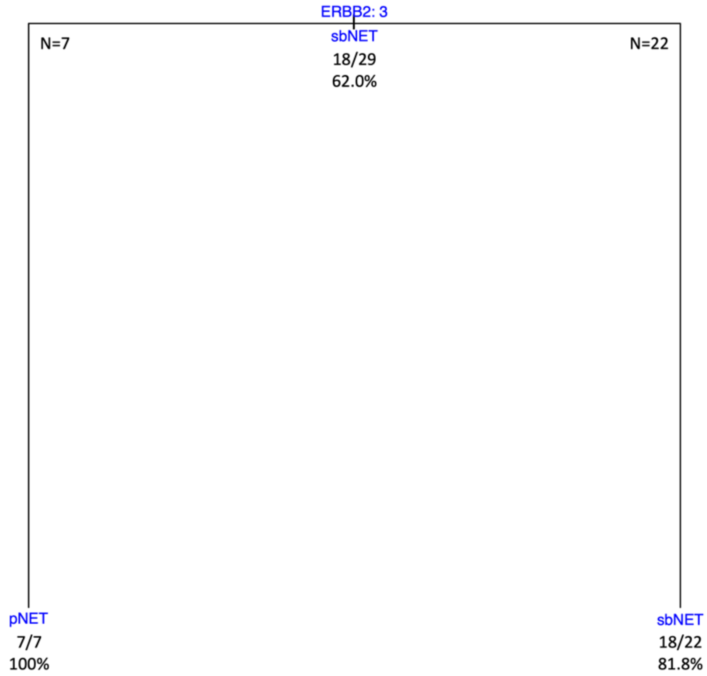
ERBB2: 3
sbNET
18/29
62.0%
N=7
N=22
pNET
7/7
100%
sbNET
18/22
81.8%

(c)

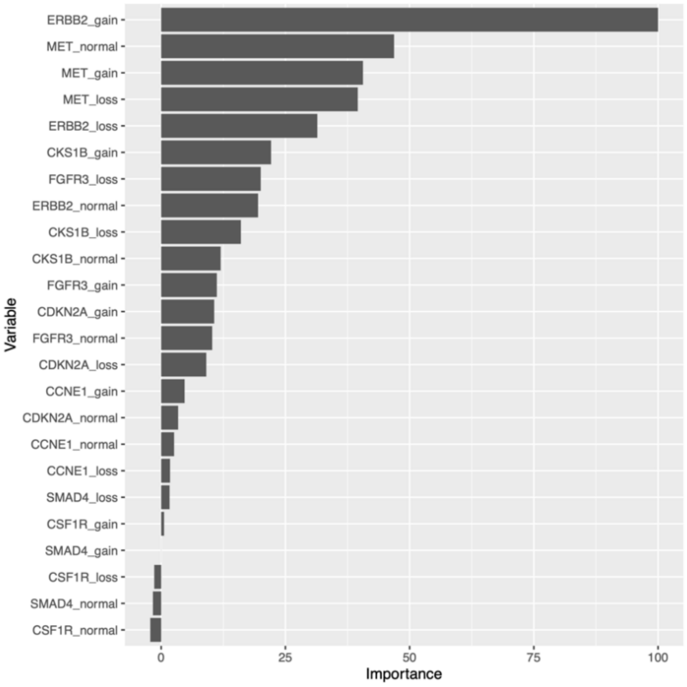

(d)

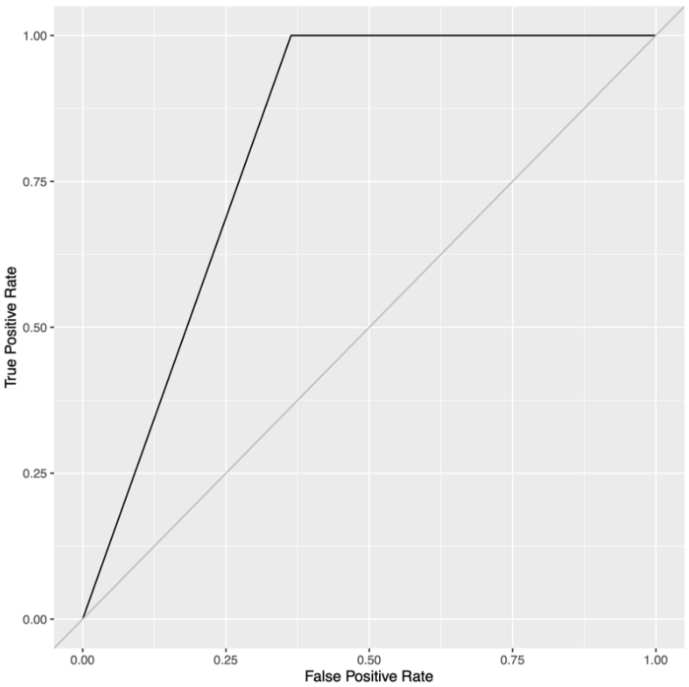

Supplementary Figure S8. DT model trained on KNN-imputed data with a transformation of the data to single categorical variables for each biomarker. Training and test sets constructed with random 80/20 dataset split (Training and Test Set 8) for manual Monte Carlo CV. (**a**) Training set DT model plot with internal nodes shown with variables being split with splitting criteria of 1 = loss, 2 = normal, and 3 = gain for number of copies at the genomic regions. Samples with a variable value equal to the splitting criteria are split down the left branch and values different than the splitting criteria are split down the right branch. Terminal nodes show the final classification of the samples with either pNET or sbNET. Classification of sample subsets at internal nodes are shown under horizontal lines and sample numbers of subsets from the split are displayed at the edges of the horizontal lines. Each node lists correctly classified samples as a fraction and percent accuracy. Tree accuracy = 74.8% (86/115) (**b**) Test set DT model plot with same design as (a) with test set data. Tree accuracy = 86.2% (25/29) (**c**) Permutation-based variable importance. All variables included in the model are assessed. Permutations per variable were performed 25 times and performance is assessed based on mean brier score. (**d**) ROC curve. AUROC curve = 0.818.

(a)

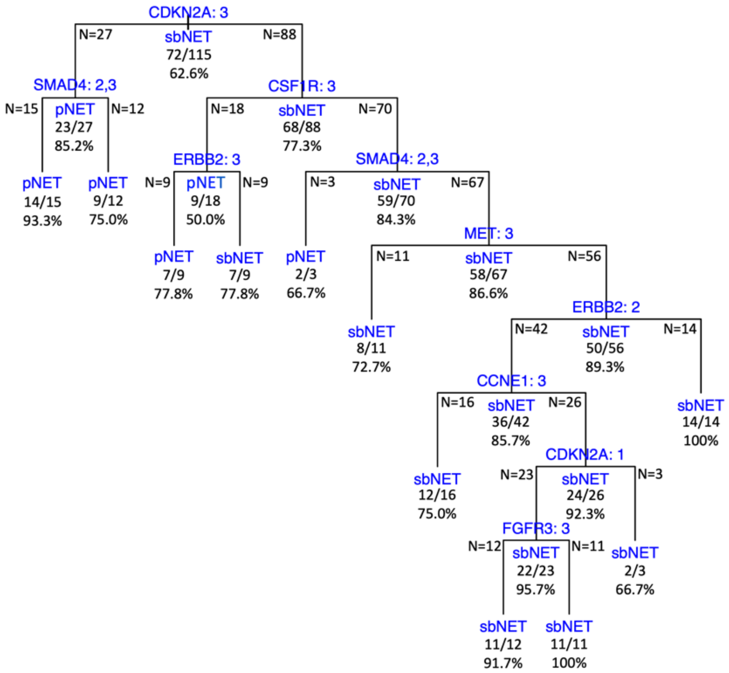

CDKN2A: 3
N=27
sbNET
72/115
62.6%
N=88
SMAD4: 2,3
N=15
pNET
23/27
85.2%
N=12
pNET
14/15
93.3%
pNET
9/12
75.0%
CSF1R: 3
N=18
sbNET
68/88
77.3%
N=70
ERBB2: 3
N=9
pNET
9/18
50.0%
N=9
pNET
7/9
77.8%
sbNET
7/9
77.8%
SMAD4: 2,3
N=3
sbNET
59/70
84.3%
N=67
pNET
2/3
66.7%
MET: 3
N=11
sbNET
58/67
86.6%
N=56
sbNET
8/11
72.7%
ERBB2: 2
N=42
sbNET
50/56
89.3%
N=14
sbNET
14/14
100%
CCNE1: 3
N=16
sbNET
36/42
85.7%
N=26
sbNET
12/16
75.0%
CDKN2A: 1
N=23
sbNET
24/26
92.3%
N=3
sbNET
2/3
66.7%
FGFR3: 3
N=12
sbNET
22/23
95.7%
N=11
sbNET
11/12
91.7%
sbNET
11/11
100%

(b)

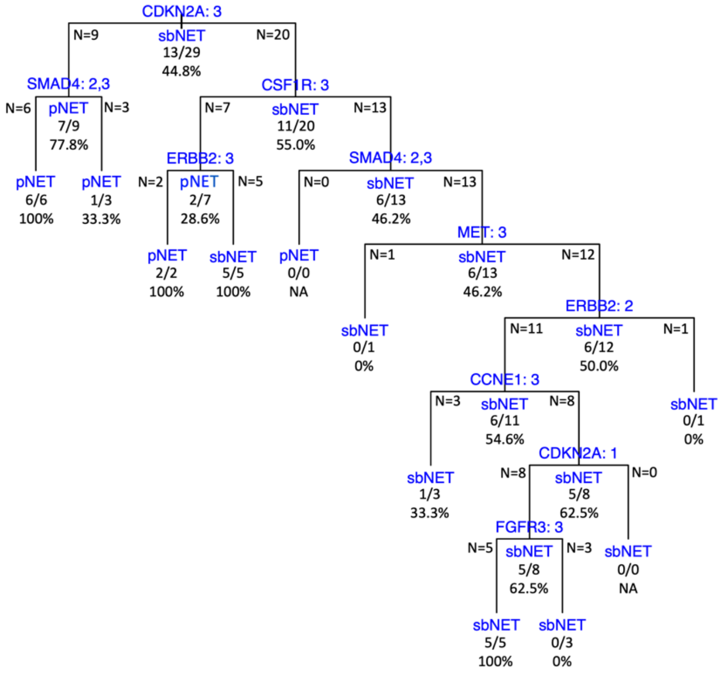

CDKN2A: 3
N=9
sbNET
13/29
44.8%
N=20
SMAD4: 2,3
N=6
pNET
7/9
77.8%
N=3
pNET
6/6
100%
pNET
1/3
33.3%
CSF1R: 3
N=7
sbNET
11/20
55.0%
N=13
ERBB2: 3
N=2
pNET
2/7
28.6%
N=5
pNET
2/2
100%
sbNET
5/5
100%
SMAD4: 2,3
N=0
sbNET
6/13
46.2%
N=13
pNET
0/0
NA
MET: 3
N=1
sbNET
6/13
46.2%
N=12
sbNET
0/1
0%
ERBB2: 2
N=11
sbNET
6/12
50.0%
N=1
sbNET
0/1
0%
CCNE1: 3
N=3
sbNET
6/11
54.6%
N=8
sbNET
1/3
33.3%
CDKN2A: 1
N=8
sbNET
5/8
62.5%
N=0
sbNET
0/0
NA
FGFR3: 3
N=5
sbNET
5/8
62.5%
N=3
sbNET
5/5
100%
sbNET
0/3
0%

(c)

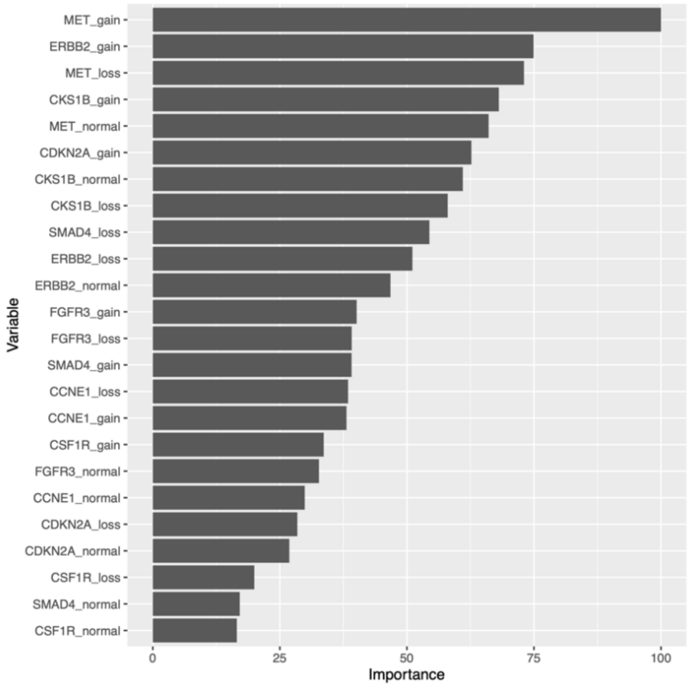

(d)

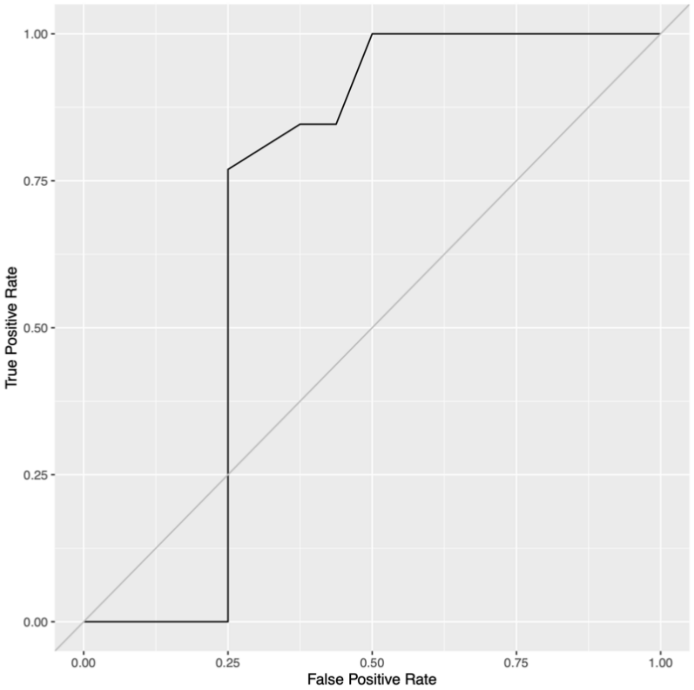

Supplementary Figure S9. DT model trained on KNN-imputed data with a transformation of the data to single categorical variables for each biomarker. Training and test sets constructed with random 80/20 dataset split (Training and Test Set 9) for manual Monte Carlo CV. (**a**) Training set DT model plot with internal nodes shown with variables being split with splitting criteria of 1 = loss, 2 = normal, and 3 = gain for number of copies at the genomic regions. Samples with a variable value equal to the splitting criteria are split down the left branch and values different than the splitting criteria are split down the right branch. Terminal nodes show the final classification of the samples with either pNET or sbNET. Classification of sample subsets at internal nodes are shown under horizontal lines and sample numbers of subsets from the split are displayed at the edges of the horizontal lines. Each node lists correctly classified samples as a fraction and percent accuracy. Tree accuracy = 84.4% (97/115) (**b**) Test set DT model plot with same design as (a) with test set data. Tree accuracy = 69.0% (20/29) (**c**) Permutation-based variable importance. All variables included in the model are assessed. Permutations per variable were performed 25 times and performance is assessed based on mean brier score. (**d**) ROC curve. AUROC curve = 0.712.

(**a**)

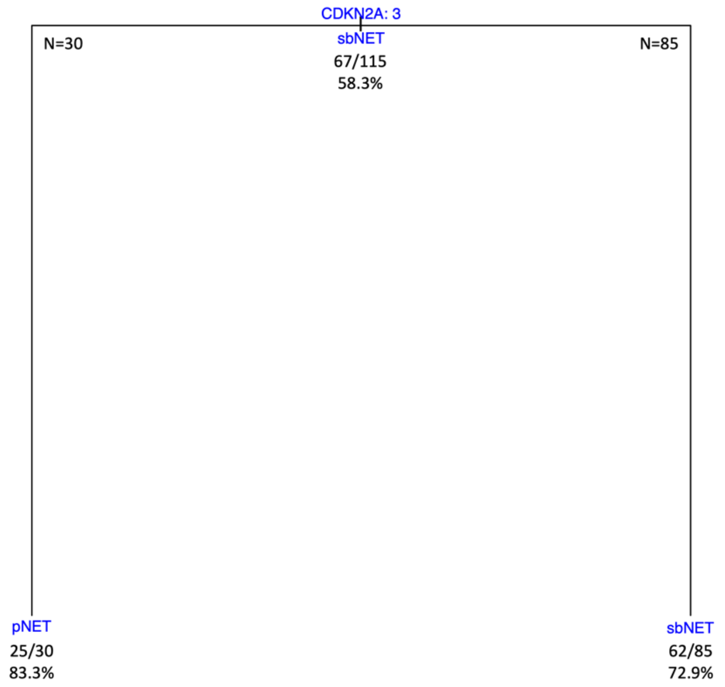
CDKN2A: 3
sbNET
67/115
58.3%
N=30
N=85
pNET
25/30
83.3%
sbNET
62/85
72.9%

(**b**)

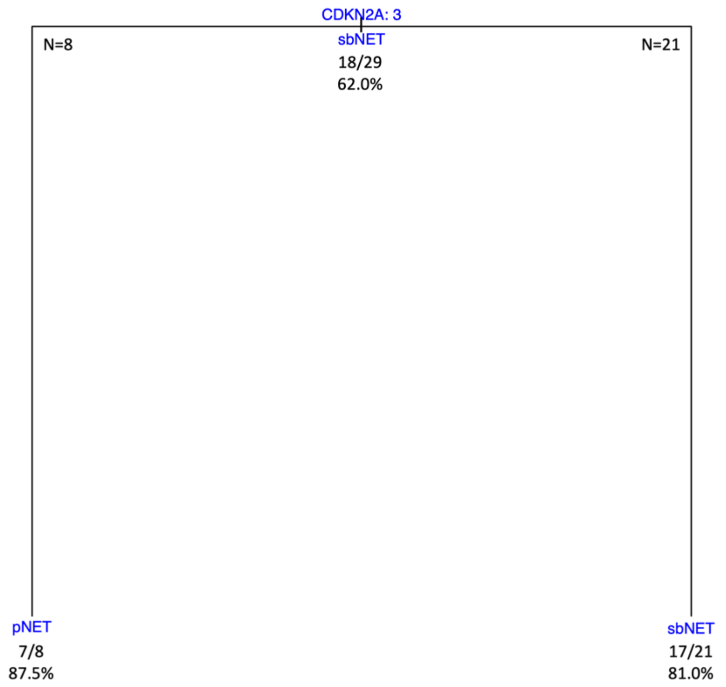
CDKN2A: 3
sbNET
18/29
62.0%
N=8
N=21
pNET
7/8
87.5%
sbNET
17/21
81.0%

(**c**)

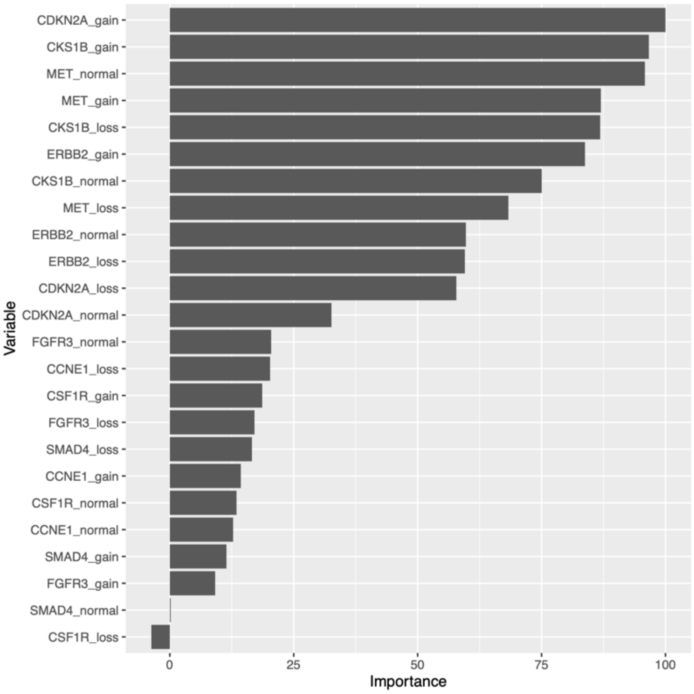

(**d**)

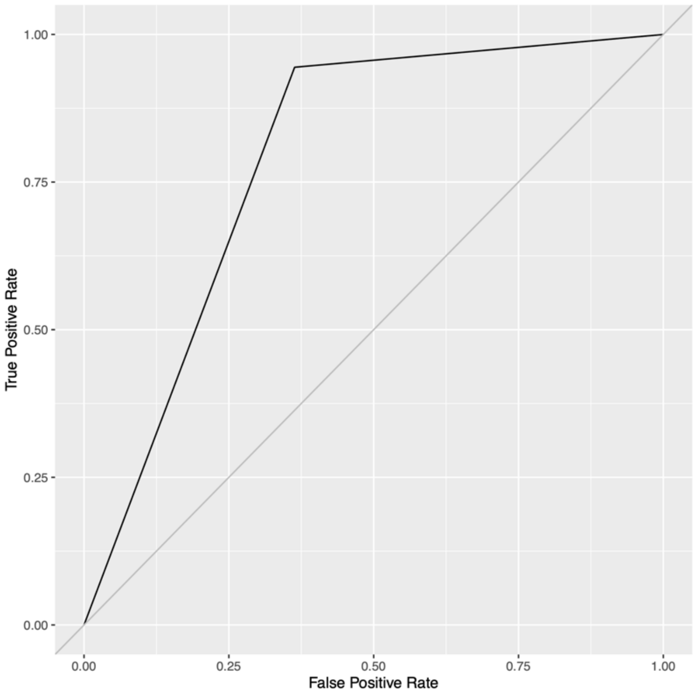

Supplementary Figure S10. DT model trained on KNN-imputed data with a transformation of the data to single categorical variables for each biomarker. Training and test sets constructed with random 80/20 dataset split (Training and Test Set 10) for manual Monte Carlo CV. (**a**) Training set DT model plot with internal nodes shown with variables being split with splitting criteria of 1 = loss, 2 = normal, and 3 = gain for number of copies at the genomic regions. Samples with a variable value equal to the splitting criteria are split down the left branch and values different than the splitting criteria are split down the right branch. Terminal nodes show the final classification of the samples with either pNET or sbNET. Classification of sample subsets at internal nodes are shown under horizontal lines and sample numbers of subsets from the split are displayed at the edges of the horizontal lines. Each node lists correctly classified samples as a fraction and percent accuracy. Tree accuracy = 75.7% (87/115) (**b**) Test set DT model plot with same design as (a) with test set data. Tree accuracy = 82.8% (24/29) (**c**) Permutation-based variable importance. All variables included in the model are assessed. Permutations per variable were performed 25 times and performance is assessed based on mean brier score. (**d**) ROC curve. AUROC curve = 0.790.

Supplementary Table S8. Counts of biomarker variables included in ten DT models from the manual Monte Carlo CV.

	CCNE1	*FGFR3*	*CSF1R*	*SMAD4*	*ERBB2*	*CKS1B*	*MET*	*CDKN2A*	Total Splits	Terminal Nodes
Model 1	1	1	1	0	2	0	1	0	6	7
Model 2	1	1	0	0	1	1	1	0	5	6
Model 3	1	1	0	2	1	1	0	1	7	8
Model 4	0	0	1	1	1	0	0	0	3	4
Model 5	0	0	1	0	1	0	0	1	3	4
Model 6	2	2	1	2	1	2	1	1	12	13
Model 7	0	0	0	0	1	0	0	0	1	2
Model 8	0	0	0	0	1	0	0	0	1	2
Model 9	1	1	1	2	2	0	1	2	10	11
Model 10	0	0	0	0	0	0	0	1	1	2
Total Count	6	6	5	7	11	4	4	6	49	59

Supplementary Table S9. DT model tables for each of the ten random 80/20 dataset splits for manual Monte Carlo CV and full dataset DT model. Terminal node metrics of training data with model performances.

Decision Tree Training Set 1 Metrics			
Probability	Prediction	Branch of Decisions	Number of Samples (Total=115)
1.000	sbNET	*ERBB2* Loss/Normal, *CSF1R* Gain/Loss, *CCNE1* Gain, *ERBB2* Loss	9/115
1.000	sbNET	*ERBB2* Loss/Normal, *CSF1R* Gain/Loss, *CCNE1* Loss/Normal	14/115
0.844	pNET	*ERBB2* Gain	32/115
0.800	sbNET	*ERBB2* Loss/Normal, *CSF1R* Normal, *FGFR3* Gain/Loss, *MET* Loss/Normal	30/115
0.625	sbNET	*ERBB2* Loss/Normal, *CSF1R* Gain/Loss, *CCNE1* Gain, *ERBB2* Gain/Normal	8/115
0.600	sbNET	Majority Class	69/115
0.563	sbNET	*ERBB2* Loss/Normal, *CSF1R* Normal, *FGFR3* Normal	16/115
0.500	pNET	*ERBB2* Loss/Normal, *CSF1R* Normal, *FGFR3* Gain/Loss, *MET* Gain	6/115

	Brier Score	Accuracy	Kappa	AUROC Curve	Sensitivity	Specificity
Tree Performance Metrics	0.142	79.1%	0.552	0.862	0.884	0.652
10-Fold CV Tuning Performance Metrics	0.180	78.2%	0.524	0.781	0.883	0.630
Held Out Test Performance Metrics	0.157	82.8%	0.654	0.841	0.813	0.846

Decision Tree Training Set 2 Metrics			
Probability	Prediction	Branch of Decisions	Number of Samples (Total=115)
1.000	sbNET	*ERBB2* Loss/Normal, *FGFR3* Loss/Gain, *MET* Loss/Normal, *CCNE1* Loss/Gain, *CKS1B* Loss/Gain	21/115
0.833	sbNET	*ERBB2* Loss/Normal, *FGFR3* Loss/Gain, *MET* Loss/Normal, *CCNE1* Loss/Gain, *CKS1B* Normal	12/115
0.811	pNET	*ERBB2* Gain	37/115
0.750	sbNET	*ERBB2* Loss/Normal, *FGFR3* Loss/Gain, *MET* Loss/Normal, *CCNE1* Normal	16/115
0.600	sbNET	*ERBB2* Loss/Normal, *FGFR3* Loss/Gain, *MET* Gain	10/115
0.583	sbNET	Majority Class	67/115
0.579	sbNET	*ERBB2* Loss/Normal, *FGFR3* Normal	19/115

	Brier Score	Accuracy	Kappa	AUROC Curve	Sensitivity	Specificity
Tree Performance Metrics	0.151	78.3%	0.538	0.847	0.896	0.625
10-Fold CV Tuning Performance Metrics	0.178	79.3%	0.562	0.787	0.881	0.670
Held Out Test Performance Metrics	0.200	72.4%	0.393	0.710	0.833	0.546

Decision Tree Training Set 3 Metrics			
Probability	Prediction	Branch of Decisions	Number of Samples (Total=115)
1.000	pNET	*ERBB2* Gain, *SMAD4* Normal/Gain	7/115
1.000	sbNET	*ERBB2* Loss/Normal, *SMAD4* Loss, *CDKN2A* Loss, *FGFR3* Loss/Gain, *CKS1B* Loss/Gain, *CCNE1* Loss/Gain	17/115
0.846	sbNET	*ERBB2* Loss/Normal, *SMAD4* Loss, *CDKN2A* Loss, *FGFR3* Loss/Gain, *CKS1B* Loss/Gain, *CCNE1* Normal	13/115
0.800	pNET	*ERBB2* Loss/Normal, *SMAD4* Normal/Gain	5/115
0.750	sbNET	*ERBB2* Loss/Normal, *SMAD4* Loss, *CDKN2A* Loss, *FGFR3* Loss/Gain, *CKS1B* Normal	12/115
0.720	pNET	*ERBB2* Gain, *SMAD4* Loss	25/115
0.654	sbNET	*ERBB2* Loss/Normal, *SMAD4* Loss, *CDKN2A* Normal/Gain	26/115
0.600	sbNET	*ERBB2* Loss/Normal, *SMAD4* Loss, *CDKN2A* Loss, *FGFR3* Normal	10/115
0.591	sbNET	Majority Class	68/115

	Brier Score	Accuracy	Kappa	AUROC Curve	Sensitivity	Specificity
Tree Performance Metrics	0.157	77.4%	0.516	0.835	0.882	0.617
10-Fold CV Tuning Performance Metrics	0.228	63.5%	0.250	0.696	0.662	0.595
Held Out Test Performance Metrics	0.104	89.7%	0.784	0.909	0.941	0.833

Decision Tree Training Set 4 Metrics			
Probability	Prediction	Branch of Decisions	Number of Samples (Total=115)
0.923	pNET	*ERBB2* Gain, *SMAD4* Normal/Gain	26/115
0.923	sbNET	*ERBB2* Loss/Normal, *CSF1R* Loss/Gain	26/115
0.706	sbNET	*ERBB2* Loss/Normal, *CSF1R* Normal	51/115
0.583	pNET	*ERBB2* Gain, *SMAD4* Loss	12/115
0.583	sbNET	Majority Class	67/115

	Brier Score	Accuracy	Kappa	AUROC Curve	Sensitivity	Specificity
Tree Performance Metrics	0.150	79.1%	0.558	0.832	0.896	0.646
10-Fold CV Tuning Performance Metrics	0.161	80.0%	0.568	0.822	0.895	0.665
Held Out Test Performance Metrics	0.169	79.3%	0.545	0.753	0.889	0.636

Decision Tree Training Set 5 Metrics			
Probability	Prediction	Branch of Decisions	Number of Samples (Total=115)
0.919	sbNET	*ERBB2* Loss/Normal, *CSF1R* Loss/Gain	37/115
0.828	pNET	*ERBB2* Gain	29/115
0.790	sbNET	*ERBB2* Loss/Normal, *CSF1R* Normal, *CDKN2A* Loss	38/115
0.644	sbNET	Majority Class	74/115
0.546	pNET	*ERBB2* Loss/Normal, *CSF1R* Normal, *CDKN2A* Normal/Gain	11/115

	Brier Score	Accuracy	Kappa	AUROC Curve	Sensitivity	Specificity
Tree Performance Metrics	0.139	81.7%	0.600	0.843	0.865	0.732
10-Fold CV Tuning Performance Metrics	0.194	77.3%	0.488	0.728	0.861	0.615
Held Out Test Performance Metrics	0.131	82.8%	0.627	0.856	0.727	0.889

Decision Tree Training Set 6 Metrics			
Probability	Prediction	Branch of Decisions	Number of Samples (Total=115)
1.000	pNET	*ERBB2* Gain, *CDKN2A* Normal/Gain, *SMAD4* Loss	6/115
1.000	sbNET	*ERBB2* Loss, *SMAD4* Loss, *CSF1R* Loss/Normal, *FGFR3* Normal/Gain, *CCNE1* Normal/Gain, *CKS1B* Loss/Gain, *CCNE1* Loss/Gain	6/115
1.000	sbNET	*ERBB2* Loss, *SMAD4* Loss, *CSF1R* Loss/Normal, *FGFR3* Normal/Gain, *CCNE1* Loss	5/115
1.000	sbNET	*ERBB2* Loss, *SMAD4* Loss, *CSF1R* Loss/Normal, *FGFR3* Loss, *MET* Normal/Gain	10/115
1.000	sbNET	*ERBB2* Loss, *SMAD4* Loss, *CSF1R* Gain, *CKS1B* Loss/Gain	12/115
0.947	pNET	*ERBB2* Gain, *CDKN2A* Normal/Gain, *SMAD4* Normal/Gain	19/115

0.833	sbNET	*ERBB2* Loss, *SMAD4* Loss, *CSF1R* Loss/Normal, *FGFR3* Loss, *MET* Loss	6/115
0.800	sbNET	*ERBB2* Loss, *SMAD4* Loss, *CSF1R* Gain, *CKS1B* Normal	5/115
0.625	pNET	*ERBB2* Loss, *SMAD4* Normal/Gain	8/115
0.574	sbNET	Majority Class	66/115
0.571	pNET	*ERBB2* Loss, *SMAD4* Loss, *CSF1R* Loss/Normal, *FGFR3* Normal/Gain, *CCNE1* Normal/Gain, *CKS1B* Normal, *FGFR3* Loss/Gain	7/115
0.571	sbNET	*ERBB2* Loss, *SMAD4* Loss, *CSF1R* Loss/Normal, *FGFR3* Normal/Gain, *CCNE1* Normal/Gain, *CKS1B* Loss/Gain, *CCNE1* Normal	14/115
0.546	sbNET	*ERBB2* Loss, *SMAD4* Loss, *CSF1R* Loss/Normal, *FGFR3* Normal/Gain, *CCNE1* Normal/Gain, *CKS1B* Normal, *FGFR3* Normal	11/115
0.500	pNET	*ERBB2* Gain, *CDKN2A* Loss	6/115

	Brier Score	Accuracy	Kappa	AUROC Curve	Sensitivity	Specificity
Tree Performance Metrics	0.120	80.0%	0.588	0.904	0.849	0.735
10-Fold CV Tuning Performance Metrics	0.164	80.4%	0.600	0.828	0.838	0.760
Held Out Test Performance Metrics	0.138	79.3%	0.582	0.895	0.737	0.900

Decision Tree Training Set 7 Metrics

Probability	Prediction	Branch of Decisions	Number of Samples (Total=115)
0.816	pNET	*ERBB2* Gain	38/115
0.714	sbNET	*ERBB2* Loss/Normal	77/115
0.539	sbNET	Majority Class	62/115

	Brier Score	Accuracy	Kappa	AUC ROC	Sensitivity	Specificity
Tree Performance Metrics	0.186	74.8%	0.482	0.736	0.887	0.585
10-Fold CV Tuning Performance Metrics	0.192	74.4%	0.468	0.729	0.888	0.570
Held Out Test Performance Metrics	0.129	89.7%	0.703	0.873	0.913	0.833

Decision Tree Training Set 8 Metrics

Probability	Prediction	Branch of Decisions	Number of Samples (Total=115)
0.771	pNET	*ERBB2* Gain	35/115
0.738	sbNET	*ERBB2* Loss/Normal	80/115
0.583	sbNET	Majority Class	67/115

	Brier Score	Accuracy	Kappa	AUROC Curve	Sensitivity	Specificity
Tree Performance Metrics	0.188	74.8%	0.461	0.722	0.881	0.563

10-Fold CV Tuning Performance Metrics	0.181	76.3%	0.499	0.765	0.864	0.625
Held Out Test Performance Metrics	0.130	86.2%	0.685	0.818	1.000	0.636

Decision Tree Training Set 9 Metrics

Probability	Prediction	Branch of Decisions	Number of Samples (Total=115)
1.000	sbNET	*CDKN2A* Loss/Normal, *CSF1R* Loss/Normal, *SMAD4* Loss, *MET* Loss/Normal, *ERBB2* Normal, *CCNE1* Loss/Normal, *CDKN2A* Loss, *FGFR3* Loss/Normal	11/115
1.000	sbNET	*CDKN2A* Loss/Normal, *CSF1R* Loss/Normal, *SMAD4* Loss, *MET* Loss/Normal, *ERBB2* Loss/Gain	14/115
0.933	pNET	*CDKN2A* Gain, *SMAD4* Nomal/Gain	15/115
0.917	sbNET	*CDKN2A* Loss/Normal, *CSF1R* Loss/Normal, *SMAD4* Loss, *MET* Loss/Normal, *ERBB2* Normal, *CCNE1* Loss/Normal, *CDKN2A* Loss, *FGFR3* Gain	12/115
0.778	pNET	*CDKN2A* Loss/Normal, *CSF1R* Gain, *ERBB2* Gain	9/115
0.778	sbNET	*CDKN2A* Loss/Normal, *CSF1R* Gain, *ERBB2* Loss/Normal	9/115
0.750	pNET	*CDKN2A* Gain, *SMAD4* Loss	12/115
0.750	sbNET	*CDKN2A* Loss/Normal, *CSF1R* Loss/Normal, *SMAD4* Loss, *MET* Loss/Normal, *ERBB2* Normal, *CCNE1* Gain	16/115
0.727	sbNET	*CDKN2A* Loss/Normal, *CSF1R* Loss/Normal, *SMAD4* Loss, *MET* Gain	11/115
0.667	pNET	*CDKN2A* Loss/Normal, *CSF1R* Loss/Normal, *SMAD4* Normal/Gain	3/115
0.667	sbNET	*CDKN2A* Loss/Normal, *CSF1R* Loss/Normal, *SMAD4* Loss, *MET* Loss/Normal, *ERBB2* Normal, *CCNE1* Loss/Normal, *CDKN2A* Normal/Gain	3/115
0.626	sbNET	Majority Class	72/115

	Brier Score	Accuracy	Kappa	AUROC Curve	Sensitivity	Specificity
Tree Performance Metrics	0.119	84.4%	0.659	0.897	0.903	0.744
10-Fold CV Tuning Performance Metrics	0.171	76.6%	0.482	0.796	0.850	0.625
Held Out Test Performance Metrics	0.252	69.0%	0.394	0.712	0.846	0.563

Decision Tree Training Set 10 Metrics

Probability	Prediction	Branch of Decisions	Number of Samples (Total=115)
0.833	pNET	*CDKN2A* Gain	30/115
0.729	sbNET	*CDKN2A* Loss/Normal	85/115
0.583	sbNET	Majority Class	67/115

	Brier Score	Accuracy	Kappa	AUROC Curve	Sensitivity	Specificity

Tree Performance Metrics	0.182	75.7%	0.471	0.723	0.925	0.521
10-Fold CV Tuning Performance Metrics	0.201	72.9%	0.419	0.707	0.838	0.575
Held Out Test Performance Metrics	0.147	82.8%	0.613	0.790	0.944	0.636

Full Dataset Decision Tree Training Set Metrics			
Probability	Prediction	Branch of Decisions	Number of Samples (Total=144)
0.897	pNET	*ERBB2* Gain, *CDKN2A* Gain	29/144
0.800	pNET	*ERBB2* Loss/Normal, *SMAD4* Normal/Gain	5/144
0.796	sbNET	*ERBB2* Loss/Normal, *SMAD4* Loss	93/144
0.590	sbNET	Majority Class	85/144
0.588	pNET	*ERBB2* Gain, *CDKN2A* Loss/Normal	17/144

	Brier Score	Accuracy	Kappa	AUROC Curve	Sensitivity	Specificity
Tree Performance Metrics	0.158	79.2%	0.560	0.793	0.871	0.678
10-Fold CV Tuning Performance Metrics	0.161	79.8%	0.571	0.800	0.893	0.663

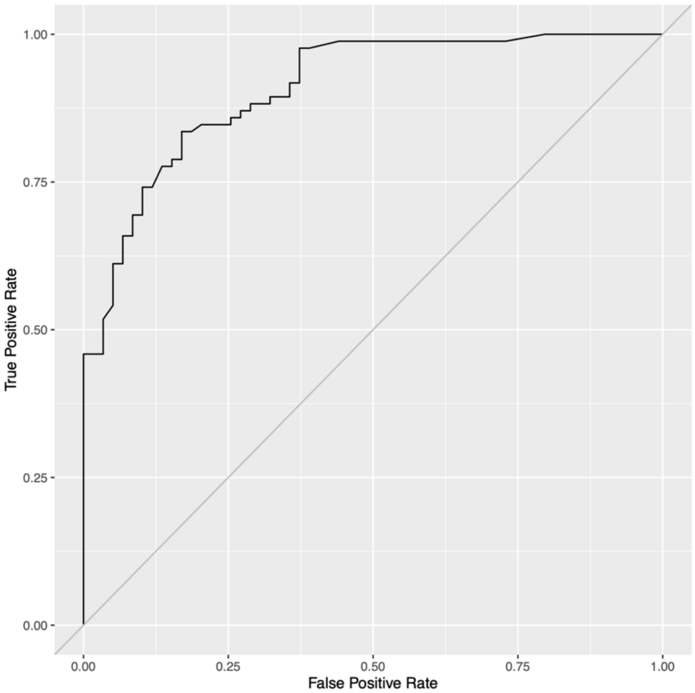

Supplementary Figure S11. Consensus tree of manual Monte Carlo CV ten DT models ROC curve. AUROC curve = 0.908.

Supplementary Table S10. Consensus tree of manual Monte Carlo CV ten DT models performance metrics.

**Brier Score**	**Accuracy**	**Kappa**	**AUROC Curve**	**Sensitivity**	**Specificity**
0.135	79.2%	0.556	0.908	0.894	0.644

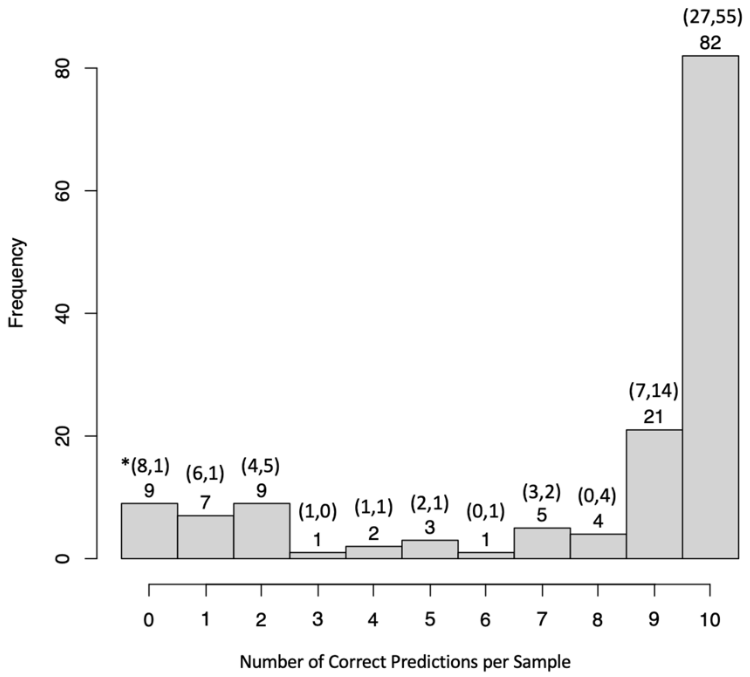

Supplementary Figure S12. Histogram of the count of samples correctly classified by the ten DT models from manual Monte Carlo CV analysis. *(Number of pNET samples, Number of sbNET samples in the subset).
